# The Microtubule Regulatory Protein Stathmin Is Required to Maintain the Integrity of Axonal Microtubules in *Drosophila*

**DOI:** 10.1371/journal.pone.0068324

**Published:** 2013-06-26

**Authors:** Jason E. Duncan, Nikki K. Lytle, Alfredo Zuniga, Lawrence S. B. Goldstein

**Affiliations:** 1 Department of Biology, Willamette University, Salem, Oregon, United States of America; 2 Howard Hughes Medical Institute, Department of Cellular and Molecular Medicine, School of Medicine, University of California San Diego, La Jolla, California, United States of America; Brigham and Women's Hospital, Harvard Medical School, United States of America

## Abstract

Axonal transport, a form of long-distance, bi-directional intracellular transport that occurs between the cell body and synaptic terminal, is critical in maintaining the function and viability of neurons. We have identified a requirement for the stathmin (*stai*) gene in the maintenance of axonal microtubules and regulation of axonal transport in *Drosophila* . The *stai* gene encodes a cytosolic phosphoprotein that regulates microtubule dynamics by partitioning tubulin dimers between pools of soluble tubulin and polymerized microtubules, and by directly binding to microtubules and promoting depolymerization. Analysis of *stai* function in *Drosophila* , which has a single *stai* gene, circumvents potential complications with studies performed in vertebrate systems in which mutant phenotypes may be compensated by genetic redundancy of other members of the *stai* gene family. This has allowed us to identify an essential function for *stai* in the maintenance of the integrity of axonal microtubules. In addition to the severe disruption in the abundance and architecture of microtubules in the axons of *stai* mutant *Drosophila* , we also observe additional neurological phenotypes associated with loss of *stai* function including a posterior paralysis and tail-flip phenotype in third instar larvae, aberrant accumulation of transported membranous organelles in *stai* deficient axons, a progressive bang-sensitive response to mechanical stimulation reminiscent of the class of *Drosophila* mutants used to model human epileptic seizures, and a reduced adult lifespan. Reductions in the levels of Kinesin-1, the primary anterograde motor in axonal transport, enhance these phenotypes. Collectively, our results indicate that *stai* has an important role in neuronal function, likely through the maintenance of microtubule integrity in the axons of nerves of the peripheral nervous system necessary to support and sustain long-distance axonal transport.

## Introduction

The organized transport of organelles, vesicles and macromolecular protein complexes is necessary to support cellular growth, function, and viability. The requirement for an efficient transport system is pronounced in neurons because their architecture typically comprises a long, narrow axon that extends to the synaptic terminal, and is orders of magnitude longer than the diameter of the cell body. The axon serves to transmit action potentials between the cell body and synaptic terminal, but also acts as a conduit for the long-distance transport of materials between these two discrete cellular compartments.

In axons, the motor proteins cytoplasmic dynein and kinesin bidirectionally transport cargo along tracks of microtubules (MTs), the main cytoskeletal component of axons. MTs are hollow polymers formed by the lateral association of linear, polarized protofilaments of heterodimers of α and β-tubulin joined head-to-tail. MTs are dynamic structures, the assembly-favored ‘plus’ ends stochastically cycling between phases of growth and shrinkage, in a process known as ‘dynamic instability’ [1]. MTs are subject to intense regulation by a vast array of factors including MT-stabilizing proteins, MT-polymerizing and depolymerizing proteins, and MT-severing proteins that act coordinately to modulate MT dynamics by directly interacting with and modifying MTs (reviewed in [2]).

Impairment of the axonal transport system is believed to cause or dramatically contribute to the development of human neurodegenerative diseases (reviewed in [3,4]). Indeed, mutations in genes that encode MT motor proteins that power axonal transport have been directly linked to human neurodegenerative diseases [5–8]. However, maintenance of the MT network that supports axonal transport is also critical. Mutations in genes encoding microtubule-associated proteins (MAPs) or proteins that regulate MT dynamics also impair axonal transport and cause human neurodegenerative disease. The neuronal MAP tau is the major constituent of insoluble intracellular inclusions called neurofibrillary tangles, a pathological hallmark of neurodegenerative tauopathies including Alzheimer’s disease. Pathogenic forms of tau have been demonstrated to impair kinesin-dependent axonal transport [9,10], and reductions in kinesin transport exacerbate neurodegeneration in animal models of tauopathies [11]. In addition, mutations in the gene spastin, which encodes a MT severing protein, are the cause of the most common form of hereditary spastic paraplegia in humans [12]. Deletion or mutation of the mouse spastin gene impairs axonal transport, resulting in progressive axonal degeneration [13,14].

We have carried out genetic screens to identify novel genes required for axonal transport. Among the candidate genes isolated, we have identified mutant alleles in the gene that encodes the MT regulatory protein stathmin. Stathmin (also known as Oncoprotein18 (Op 18), Phosphoprotein p19, metablastin, Prosolin, and Leukemia Associated Protein 18) is a cytosolic phosphoprotein that regulates MT dynamics by partitioning tubulin dimers from pools of soluble tubulin thus preventing their assembly into polymerized MTs, and by directly binding to MTs and promoting their disassembly [15,16]. These functions are attributable to different regions of the stathmin protein. The N-terminus of stathmin promotes MT catastrophes whereas the C-terminus exhibits tubulin-sequestering activity [17]. Four genes encode the vertebrate stathmin family of proteins; STMN1 encodes the ubiquitous, cytosolic stathmin protein, while STMN2, STMN3 and STMN4 encode the neuron-specific, membrane-associated stathmin-like proteins SCG10, SCLIP and RB3/RB3'/RB3" respectively [18]. All share a homologous tubulin-binding stathmin-like domain, while SCG10, SCLIP and RB3 contain an N-terminal membrane-targeting domain [19]. The vertebrate stathmin proteins can bind two tubulin heterodimers and are subject to regulatory phosphorylation on four conserved serine residues that reduce their ability to bind tubulin and regulate MT polymerization [20,21].

In *Drosophila* , a single *stathmin* (*stai*) gene encodes four protein isoforms, representative of the entire vertebrate stathmin family [22]. The *stai* gene is differentially expressed during *Drosophila* embryogenesis, with transcripts encoding staiA isoforms maternally deposited to the embryo, whereas the staiB encoding isoforms are expressed at high levels in early neuroblasts, and later in the developing embryonic central and peripheral nervous system [22]. Neuronal enriched *staiB* isoforms have an N-terminal domain that has conserved cysteine amino acids that are potential palmitoylation sites thought to play a role in subcellular targeting of the protein [22]. Analysis of loss of *stai* function in *Drosophila* has allowed us to circumvent potential complications encountered in studies in vertebrate systems in which mutant phenotypes may be compensated by genetic redundancy of other member of the *stai* gene family.

In this study, we show through loss of *stai* activity, an essential function for *stai* in the maintenance of axonal MTs necessary to support axonal transport. The architecture of the axonal MT network appears severely disrupted in our *stai* mutants, implicating a critical role for *stai* in its regulation. Loss of *stai* activity results in a posterior paralysis and 'tail-flip' phenotype in third instar larvae, hallmarks of impaired axonal transport. Immunohistochemistry reveals aberrant accumulation of synaptic vesicles in the axons of segmental nerves of *stai* mutant larvae. Despite the disruption in axonal transport, a significant number of mutant animals survive to the adult stage but have a reduced lifespan. In addition, *stai* mutant adults exhibit a progressive, late-onset bang-sensitive seizure in response to mechanical stimulation. This epilepsy-like behavioral deficit is characteristic of the class of mechanical shock-sensitive *Drosophila* mutants that have altered neuronal excitability and are used to model human epilepsy [23]. We demonstrate by mutant analysis and transgene rescue that the observed phenotypes are the result of loss of *stai* function. We also show that expression of the human stathmin gene, STMN1, can rescue these defects indicating that the *Drosophila* and human proteins are functional orthologs. Genetic reduction in the levels of Kinesin-1, the primary anterograde motor of axonal transport, enhances *stai* mutant phenotypes. Collectively, our results indicate that stai has an important function in the maintenance of the integrity of MTs in the axons of nerve cells of the peripheral nervous system necessary to support axonal transport.

## Materials and Methods

### Fly Stocks

*Drosophila* were raised on standard culture media at 25^o^C. Strains used in these studies include OregonR, CantonS, *w* ^1118^ *, stai* ^*rdtp*^, *PBac*{*5HPw* ^*+*^} *stai* ^*B200*^, *Df*(*2L*) *BSC5*, *Df*(*2L*) *ED384*, *Df*(*2L*) *ED385*, *Df*(*2L*) *BSC239*, *Df*(*2L*) *Exel6015*, *Khc* ^20^, *w* ^1118^; *CyO* P{FRT(*w* ^*+*^) Tub-PBac\T} 2/wg^Sp-1^, tub-Gal4, and tubulin-*staiB2* (gift of Pernille Rorth, IMCB, Singapore), tubulin-*STMN1*. *PBac*{*5HPw* ^*+*^} *stai* ^*B200*^ is a *stai* mutant *Drosophila* strain obtained from the Bloomington *Drosophila* Stock Center and is derived from a progenitor line containing a 5’ half-P construct generated by Brian Ring and Dan Garza [24].

The *piggyBac* insertion in *PBac*{*5HPw* ^*+*^} *stai* ^*B200*^ was precisely excised by crossing *PBac*{*5HPw* ^*+*^} *stai* ^*B200*^/*CyO* against *w* ^1118^; *CyO* P{FRT(*w* ^*+*^) Tub-PBac\T} 2/wg^Sp-1^ and generating individual balanced stocks that were determined by eye color to have the *piggyBac* transposon excised. The nature of the excision for each was confirmed by PCR across the original insertion site and subsequent sequence analysis.

The *stai* ^*B200*^ *Khc* ^20^ chromosome was generated by meiotic recombination, and balanced over *CyO Act GFP*. Recombinant *stai* ^*B200*^ *Khc* ^20^ chromosomes were identified by outcrossing each potential recombinant line against *w* ^1118^ and following the inheritance of the mini *w* ^+^, associated with *stai* ^*B200*^, in straight-winged progeny. The presence of *Khc* ^20^ was inferred by the absence of straight-winged flies in each recombinant stock, and confirmed by lethality in heterozygous combination with *Df(2R) Jp8*.

### PCR Amplification and Sequencing of the *stai* Genomic Region

Genomic DNA was isolated from homozygous third instar *stai* ^*rdtp*^ larvae according to standard protocol [25]. The coding region of the *stai* gene was amplified in four separate PCR reactions using HotStar HiFidelity DNA Polymerase (Qiagen, Valencia, CA) according to manufacturer’s recommended protocol. PCR primers used were stai1 5’-GCTAATCAAACGTGCTTAAAGCGAATT-3’, stai2 5’-GTTTCGAATCAGTTGAGTAAGAATATA-3, stai3 5’-GCATTACTCGAATCAATCAAGTTCAA-3’, stai4 5’-GTATCCACATTCGCTGTAGATGGATCT-3’, stai5 5’-GCCAGCAATCAAACACTTCAACAATGA-3’, stai6 5’-GCATTTTATCTGCTTCATGCATAGTTG-3’, stai7 5’-CGATGTAACACTAAATTCGTATTGTTT-3’, and stai8 5’-CTCGCTCAGCGGTATCTTGAGACTTTG-3’. All PCR products were resolved by agarose gel electrophoresis and purified using the QIAquick Gel Extraction Kit (Qiagen). PCR products were sequenced by Eurofins MWG Operon (Huntsville, AL) using the primers designed to PCR amplify the *stai* locus, in addition to the oligonucleotides stai9 5’-GCTACCAAACCTATTGCATCCCATAGT-3’ and stai10 5’-GACTGGACTAGCAAACTGGTTAACATT-3’.

### qRT-PCR

mRNA purified from third instar larvae using the Oligotext Direct mRNA Mini Kit (Qiagen) was used to synthesize cDNA with the SuperScript III First-Strand Synthesis System for RT-PCR (Invitrogen, Carlsbad, CA). RT-PCR was performed with the SYBR Green PCR Master Mix (Applied Biosystems) on an ABI 7300 Real Time PCR System (Applied Biosystems). In order to distinguish between *stai* transcripts with different transcriptional start sites, *stai* message was quantified with 'forward' oligonucleotides complementary to coding sequence within exon 2 (5’- GGTGAACAACACTGTTGATACTG -3’) shared by *staiB* isoforms, and sequence downstream of the copia insertion site in *stai* ^*rdtp*^ within exon 1’ (5’- GATTAGTACGCGACTCGGTGA -3’) shared by *staiA* isoforms, in separate amplifications with a ‘reverse’ oligonucleotide complementary to sequence in exon 3 (5’- CTCAATCTCCTCGACGCTAAC -3’), shared by all *stai* isoforms. Results were normalized against the signal from the *GAPDH* gene product (5'-AATTAAGGCCAAGGTTCAGGA-3', 5'-ACCAAGAGATCAGCTTCACGA-3'). All RT-PCR experiments were repeated five times for each genotype, and analyzed and quantified using the comparative C_T_ method (ΔΔC_T_ method). The results are presented as mean ± standard deviation (SD). Significance was determined by ANOVA of ΔC_T_ values [26,27].

### Quantification of the Posterior Paralysis 'Tail-Flip' Phenotype

The posterior paralysis phenotype of late third instar larvae was analyzed as described previously [28]. Briefly, third instar larvae of each genotype were collected, rinsed in ddH_2_O and placed in 60 x 15 mm culture dishes, and the crawling behavior of each larva observed. A minimum of one hundred larvae was analyzed for each genotype. The severity of the posterior paralysis phenotype was scored and quantified using the following criteria; larvae were determined to exhibit a *robust tail-flip* if the tail was raised greater than 40^o^ above horizontal when crawling; a *mild tail-flip* if the tail was raised less than 40^o^ above horizontal when crawling, and *no-tail flip* if the larva exhibited a normal crawling behavior. The crawling behavior of third instar larvae was recorded with a Canon PowerShot G10 coupled to an Olympus SZ61 stereomicroscope and the resultant movies processed in iMovie and QuickTime Pro.

### Larval Preparation, Immunohistochemistry and Microscopy

Immunostaining of larvae was performed according to standard protocol [29]. In brief, wandering third instar larvae were dissected in Ca^2+^ free HL3.1 buffer [30] and fixed for 30 minutes in freshly prepared 4% paraformaldehyde in PBS, pH 7.4. The larvae were washed and permeabilized in PBT (PBS + 0.2% Triton X-100), blocked in 1% BSA in PBT, and incubated overnight at 4^o^C with primary antibody in PBT. Following removal of the primary antibody, the larvae were washed in PBT and incubated for 2 hours at room temperature with fluorescently conjugated secondary antibody in PBT. Following removal of the secondary antibody and PBT washes, larval preparations were mounted on glass slides in Vectashield mounting medium (Vector Laboratories, Burlingame, CA). Antibodies were used at the following concentrations; mouse monoclonal anti- *Drosophila* cysteine string protein (DCSP-2 mAb 6D6) 1:200, anti-Futsch (mAb 22c10) 1:5 (Developmental Studies Hybridoma Bank, IA), anti-α-tubulin (mAb DM1A) 1:1000 (Sigma, St. Louis, MO), anti-acetylated α-tubulin (mAb 611B1) 1:250 (Sigma), Alexa Fluor 488 goat anti-mouse 1:250, and Alexa Fluor 568 goat anti-mouse 1:250 (Invitrogen). Nuclei were visualized by co-staining with Syto24 (Invitrogen) at a 1:1000 dilution during secondary antibody treatment.

### Confocal Imaging and Analysis

All images were acquired on a Zeiss LSM-710 laser scanning confocal microscope. Imaging parameters, including signal gain, for a given experimental treatment were set such that the brightest treatment group produced images that were non-saturating. All imaging parameters were kept constant across a given experimental treatment.

Images of segmental nerve axons represent three-dimensional maximum intensity projections of serial stacks, ~10.4-14.2 µm thickness, acquired from the four to six medial segmental nerves spanning abdominal segments A4-A6. Disruptions in axonal transport were quantified from maximal intensity projection images of segmental nerve axons as they passed through abdominal segments A2 and A4 using the threshold and particle analysis functions in ImageJ (ver 1.47k) with a size detection minimum of 0.5 µm^2^. Results are reported as number of CSP accumulations/50 µm segmental nerve axon. Futsch and acetylated α-tubulin staining intensity was quantified using the histogram function of the Zen 2010 image analysis software package that reports a distribution of pixel intensities, as well as average pixel intensity, in a selected region of interest of an image. A lower threshold for the histogram analysis was applied to all images analyzed. Statistical analysis across the genotypes analyzed was performed using ANOVA of average pixel intensity of all segments analyzed within each experimental group, followed by Fisher’s LSD *post hoc* analysis.

Images of larval muscle represent three-dimensional maximum intensity projections of serial stacks, ~14.8-16.2 µm thickness, through muscle 6 at abdominal segment A4. Perinuclear MT density was quantified with ImageJ 1.44o by measuring the ratio of tubulin positive: negative staining signal in an area that extends 5 µm around the nucleus. Average perinuclear MT density was determined for each genotype by measuring MT density surrounding at least twenty nuclei, recorded from six larvae. The average perinuclear MT density for each genotype was normalized against wildtype.

### Analysis of Lifespan and Bang-Sensitive Phenotypes

The life span of male *Drosophila* was measured at 25^o^C. Females were excluded from the life span analysis due to observed sex differences regarding the effect of dietary restriction on lifespan [31]. Adult flies were collected 24-hours post eclosion and maintained in standard culture vials (25 mm x 95 mm) with a maximum of 10 flies per vial. Flies were counted daily and transferred to fresh vials every other day. A minimum of one hundred flies per genotype was analyzed. The mean lifespan for each genotype was calculated by averaging individual lifespans for flies within a cohort. Significance was determined by ANOVA followed by Fisher’s and Scheffe’s *post hoc* analysis.

The bang-sensitive phenotype was quantified by the methodology of Ganetzky and Wu [32]. Briefly, male *Drosophila* of each genotype were aged at 25^o^C. Forty-eight hours prior to testing, the aged flies were CO_2_ anaesthetized and a maximum of five flies were placed into fresh vials. The flies were mechanically stimulated by vortexing at full-speed in culture vials for 10 seconds and the number of flies exhibiting paralysis counted. The penetrance and severity of the bang-sensitive phenotype was quantified by testing a minimum of fifty aged adult male flies of each genotype for bang sensitivity following mechanical stimulation at 1, 7, 14, 21, 42 and 56 days post eclosion. In addition, the average time period required for recovery from the paralysis was also quantified for each fly by measuring the time from the end of the vortex period until the paralyzed flies regained the ability to stand and take their first steps.

### Western Blot Analysis

Five third instar larvae of each genotype were homogenized in 100 µl of 2x NuPAGE LDS Sample Buffer (Invitrogen), incubated at 80^o^C for 10 minutes, and centrifuged at 16,000 x g for 5 minutes. The supernatant was decanted into new microcentrifuge tubes and the protein concentrations determined using the RC-DC Protein Assay (Bio-Rad, Hercules, CA). For each genotype, 25 µg of protein was resolved on NuPAGE 4-12% Bis-Tris Polyacrylamide Gels and transferred to nitrocellulose membrane (0.45 µm) (Bio-Rad) at 300 mA for 120 minutes in Tris-Glycine transfer buffer (25 mM Tris-Base, 190 mM Glycine). The efficiency of transfer was confirmed by Ponceau S stain (0.1% w/v Ponceau S, 5% v/v acetic acid). The blot was incubated in blocking buffer (5% Carnation dry milk, 50 mM Tris, 150 mM NaCl, 0.5% Tween 20) for one hour at room temperature prior to overnight incubation with primary antibody in antibody dilution buffer (1% Carnation dry milk, 50 mM Tris, 150 mM NaCl, 0.5% Tween 20) at 4^o^C. Following washes in wash buffer (0.2% Carnation dry milk, 50 mM Tris, 150 mM NaCl, 0.05% Tween 20), the blot was incubated with secondary antibody for two hours at room temperature. The SuperSignal Chemiluminescent Substrate Kit for Western Blotting (Pierce, Rockford, IL) was used for detection and the blot was exposed to X-ray film and developed for signal visualization. Primary antibodies included anti-α-tubulin 1:5,000 (DM1Α T-9026, Sigma), anti-acetylated α-tubulin 1:5,000 (T-6793, Sigma), anti-actin 1:10,000 (mAb1501, Chemicon), anti-GAPDH (IMG-3073, Imgenex Corp, San Diego, CA) anti-Kinesin heavy chain 1:5,000 (AKIN01, Cytoskeleton, Denver, CO) and anti-kinesin light chain 1:1,000. Secondary antibodies included HRP conjugated Goat anti-Mouse IgG (81-6520) and HRP conjugated Goat anti-Rabbit IgG (65-6120) used at 1:20,000 dilutions (Zymed Laboratories, Invitrogen Immunodetection). Western blots were quantified and data corrected for load using anti-GAPDH antibody as a loading control. Quantitation was performed using a BioRad ChemiDoc XRS+ chemiluminescence detection system with a 16-bit CCD camera and ImageJ.

### Generation of Antisera against *Drosophila* Kinesin Light Chain

Rabbit polyclonal antisera was produced against recombinant *Drosophila* kinesin light chain protein following PCR amplification of the *Klc* gene from cDNA clone NM_079325.3 with primers Klc(Xho1) 5'-ATCCGAGCTCGAGACGCAAATGTCGCAG-3' and Klc(Kpn1) 5'-CGAATTCCATGGTACCTTATGGTTTCGC-3'. The resultant PCR product was cut with Xho1 and Kpn1 and subcloned into the Xho1/Kpn1 site of the pRSET-A plasmid to generate pRSET-A-*Klc*. pRSET-A-*Klc* was sequenced to confirm a complete *Klc* open reading frame, transformed into BL21(DE3) pLysS, and expression of the His-tagged Klc protein was induced in a 100 ml culture at OD_600_=0.5 with IPTG to a final concentration of 1 mM. The cells were harvested four hours post induction and the recombinant Klc protein was purified using nickel-nitrilotriacetic acid (Ni-NTA) agarose resin (Qiagen), electrophoretically isolated and used to raise antisera in rabbits (Covance Research Products, Denver, PA). The specificity of the Klc antibody was confirmed by western blot against total protein extract isolated from third instar larvae. In wildtype *Drosophila* , the antibody recognized a single band of approximately 60 kDa, and a reduction in the intensity of the signal from this 60 kDa band was observed in protein from a heterozygous strain harboring the chromosomal deletion *Df*(*3L*) *8ex94* that removes the *Klc* gene [33].

### Generation of Transgenic *Drosophila* for Ubiquitous Expression of Human Stathmin

PCR was used to introduce a 5' Not1 and 3' Xho1 restriction site flanking the full-length open reading frame of the cDNA clone encoding human stathmin 1 (*STMN1*) transcript variant 1 (Accession No. NM_203401.1, gift of Lynne Cassimeris, Lehigh University, PA) with PCR primers hstai(Not1) 5'-GAGCGGCCGCATGGCTTCTTCTGATATC-3' and hstai(Xho1) 5'-GACTCGAGTTAGTCAGCTTCAGTCTC-3'. The PCR product was cut with Not1 and Xho1 and directionally subcloned into the Not1/Xho1 site of the pCaSpeR-tub plasmid (gift of Pernille Rorth, IMCB, Singapore) to generate pCaSpeR-tub-*STMN1*. The pCaSpeR-*STMN1* rescue construct was sequenced to verify a complete *STMN1* open reading frame. Standard germline transformation techniques [34] were employed to generate multiple independent transgenic lines that were mapped to identify third chromosome insertion lines used to generate homozygous stocks.

## Results

### Isolation and Molecular Characterization of *stathmin* Mutant Alleles

The *redtape* (*rdtp*) mutation was isolated in a genetic screen for recessive third chromosome P-element induced mutations that disrupt axonal transport in *Drosophila melanogaster* (unpublished data). The basis for the mutagenic screen was a posterior paralysis of third instar larvae, observed as a 'tail-flip', previously described for mutations in genes that encode proteins required for axonal transport [28,29,35–38]. Initial analysis of the *rdtp* mutation led to the discovery that the posterior paralysis observed in homozygous mutant larvae was due to a second chromosome mutation and not the P-element insertion. The *rdtp* mutation was subsequently mapped to cytological region 26B5-26C1 on the left arm of the second chromosome by its failure to complement *Df*(*2L*) *BSC5*, *Df*(*2L*) *ED384*, *Df*(*2L*) *ED385*, *Df*(*2L*) *Exel6015* and *Df*(*2L*) *BSC239*. Further analysis identified *PBac*{*5HPw* ^*+*^} *stai* ^*B200*^, obtained from the Bloomington *Drosophila* Stock Center, as a non-complementing gene mutation. Heteroallelic combination of *rdtp* and *stai* ^*B200*^ recapitulated the larval ‘tail flip’ phenotype originally observed in *rdtp* homozygotes, indicating that *rdtp* is a mutant allele of the *stai* gene. The *rdtp* mutation was subsequently renamed *stai* ^*rdtp*^.

**Figure 1:**
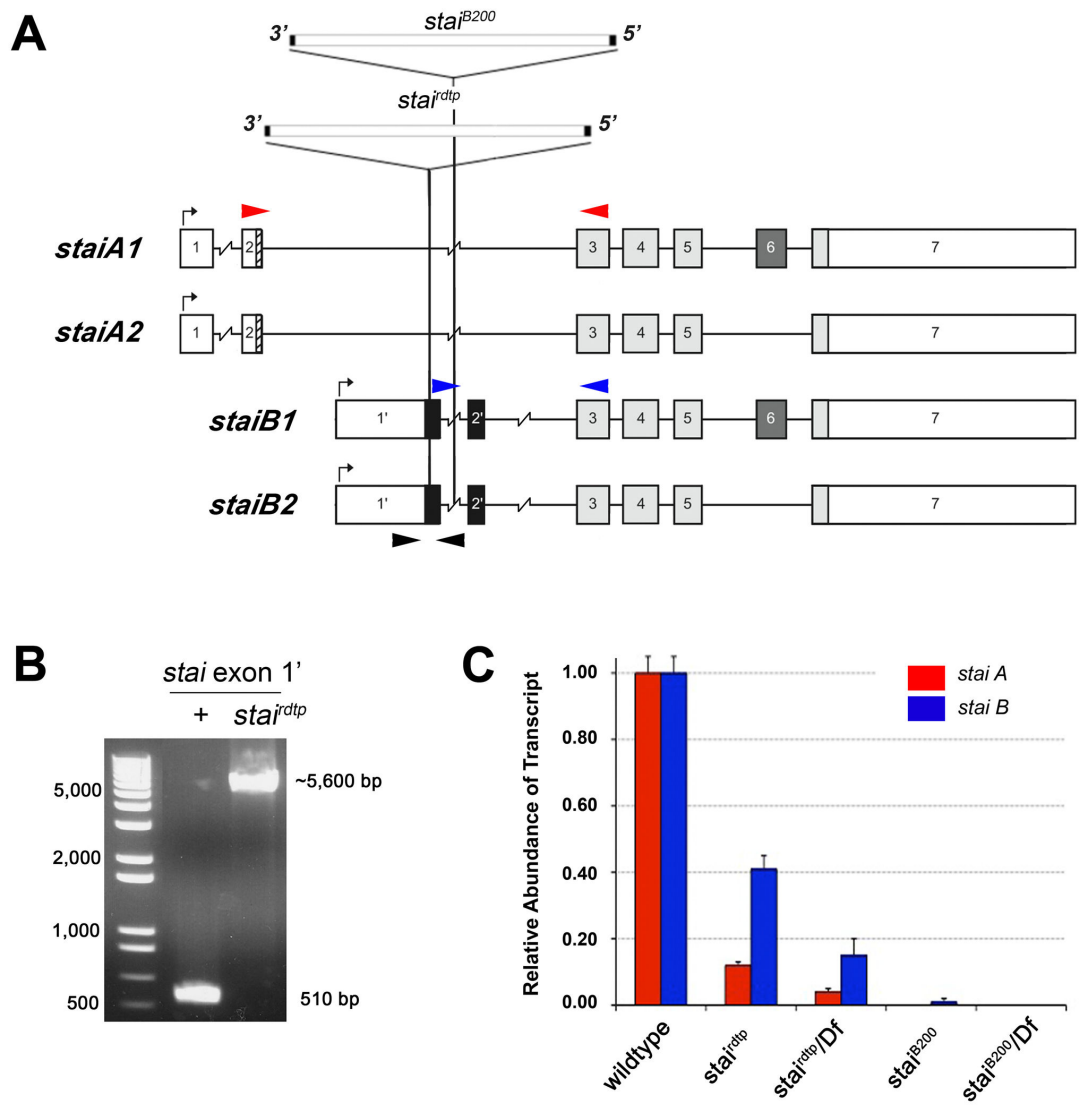
Identification of Mutations in the *stathmin* (*stai*) Locus. (**A**) Genomic structure of the *Drosophila* *stai* locus and the positions of the mutagenic copia retrotransposon *stai* ^*rdtp*^ and the *piggyBac* element *stai* ^*B200*^. Exons are boxed, noncoding portions of exons are white, exons 3, 4, 5, shared by all stai proteins, are light grey (after Lachkar et al, 2010). Transcripts encoding *staiA* isoforms include exons 1 and 2 with alternative splicing that either includes (*staiA1*) or excludes (*staiA2*) exon 6. Transcripts encoding *staiB* isoforms include exons 1’and 2’ with alternative splicing that either includes (*staiB1*) or excludes (*staiB2*) exon 6. The copia retrotransposon *stai^rdtp^* is inserted in the open reading frame of the *stai* gene in exon 1', ten base pairs downstream of the translational start site used to produce nervous system enriched *staiB* encoding transcripts. The *piggyBac* element *PBac*{*5HPw^+^*}*stai^B200^* is inserted in the 2.8 kb intron separating exons 1’ and 2’, 1.3kb downstream of the splice junction of exon 1’. (**B**) The copia insertion in the *stai* gene was identified by PCR amplification across the open reading frame of *stai* exon 1’ that resulted in an unexpectedly large 5.6 kb product from genomic DNA isolated from *stai* ^*rdtp*^ homozygous larvae. The black arrowheads in Figure 1A represent the relative position of PCR primers used. (**C**) qRT-PCR of *staiA* and *staiB* mRNA derived from third instar larvae is shown. The expression of *staiA* and *staiB* is significantly reduced in all *stai* mutant genotypes analyzed compared to wild type expression levels (P<0.01). The red and blue arrowheads in Figure 1A represent the relative position of primers used for qRT-PCR of *staiA* and *staiB* message. Results are normalized against the expression of the *GAPDH* gene product and are presented as mean ± SD.

PCR amplification of the *stai* gene from homozygous second instar *stai* ^*rdtp*^ larvae generated an ~5.6 kb PCR product for exon 1', larger than the predicted 510 bp PCR product observed in wildtype (Figure 1B). The 5.6 kb PCR product was sequenced and found to encode a copia retrotransposon, a member of a broad class of natural genetic elements characterized by long direct terminal repeats [39]. Spontaneous copia-induced mutations have been previously reported for many genes in *Drosophila* [40–44]. The copia element in *stai* ^*rdtp*^ is inserted in the open reading frame of the *stai* gene (Figure 1A). The non-complementing *stai* ^*B200*^ mutation is the result of a *piggyBac* transposon insertion in the 2.8 kb intron separating exons 1’ and 2’ of the *stai* gene (Figure 1A), 1337bp downstream of exon1’ and 1469bp upstream of exon 2’ (genome sequence position 2L:6,111,561 – FlyBase version FB2013_01, released January 23^rd^, 2013). 

### Expression of *stai* is Reduced in *stai* ^*rdtp*^ and *stai* ^*B200*^ Larvae

We used qRT-PCR to determine if the copia insertion in *stai* ^*rdtp*^ and the *piggyBac* insertion in *stai* ^*B200*^ altered the expression level of the *stai* gene in third instar larvae. Amplification with primer pairs specific for detection of *staiA* and *staiB* mRNA isoforms revealed significantly reduced levels of both transcripts in homozygous *stai* ^*rdtp*^ third instar larvae (P<0.01) (Figure 1C). Transcripts encoding *staiA* isoforms were more strongly affected, reduced to as little as 12.0 ± 1.0% (mean ± SD) of wild type levels, compared to *staiB* encoding transcripts, which were reduced to 41.0 ± 4.0% of wild type levels (Figure 1C). As expected, heterozygous combination of *stai* ^*rdtp*^ with *Df*(*2L*) *Exel6015*, a 170 kb chromosomal deletion that removes 14 genes including *stai*, reduces the abundance of *staiA* and *staiB* derived transcripts to 4.0 ± 1.0% and 15.0 ± 5.0% respectively, approximately 50% of the levels observed in *stai* ^*rdtp*^ homozygous larvae (Figure 1C). The level of *staiB* message is also reduced in homozygous *stai* ^*B200*^ mutant third instar larvae, to 1.0 ± 1.0% of wildtype (P<0.0001) (Figure 1C). The levels of *staiA* encoding transcript in homozygous *stai* ^*B200*^ larvae and message encoding both *staiA* and *staiB* isoforms in *stai* ^*B200*^ */Df*(*2L*) *Exel6015* larvae are reduced to undetectable levels (Figure 1C).

### Lethal Phase Analysis of *stai* Mutants

Despite the dramatic reduction in *stai* message, the *stai* ^*B200*^ allele causes semi-lethality, with approximately 40% of the expected homozygous progeny from a heterozygous cross surviving to the adult stage (n=1900), suggesting that *stai* function is not essential for adult viability. These results are supported by the previous observation that *stai* null *Drosophila* are adult viable [45]. Unexpectedly, homozygous *stai* ^*rdtp*^ larvae were inviable past the third instar stage with no homozygous adults recovered among the progeny of a heterozygous cross (n=1000). This was confounding given that greater *stai* message was detected in homozygous *stai* ^*rdtp*^ larvae than *stai* ^*B200*^ larvae (Figure 1C) and that *stai* null *Drosophila* are reported to be adult viable [45]. The mutagenic copia insertion in *stai* ^*rdtp*^, however, is inserted in exon 1' of the *stai* open reading frame used to produce neuronal *staiB* splice variants. We considered the possibility that the copia sequence was incorporated in the *staiB* message produced by *stai* ^*rdtp*^ mutant larvae, resulting in a dominant negative protein. However, a significant number of viable adults were observed when the *stai* ^*rdtp*^ chromosome was placed in heterozygous combination with *stai* ^*B200*^ or *Df*(*2L*) *Exel6015*. Therefore, it is likely that an additional mutation responsible for the homozygous lethality exists on the *stai* ^*rdtp*^ chromosome. Given the likely presence of a second, lethal mutation on the *stai* ^*rdtp*^ chromosome, and the observation by others that copia-induced gene mutations are subject to the action of suppressor or enhancer alleles mapping to other loci, resulting in complex phenotypes [46,47], the focus of this study is the *stai* ^*B200*^ mutant allele and the use of the *stai* ^*rdtp*^ chromosome is limited to heterozygous analysis or in heteroallelic combination with *stai* ^*B200*^ and *Df*(*2L*) *Exel6015*.

### Loss of *stai* Function Results in a Larval Posterior Paralytic Phenotype

**Figure 2:**
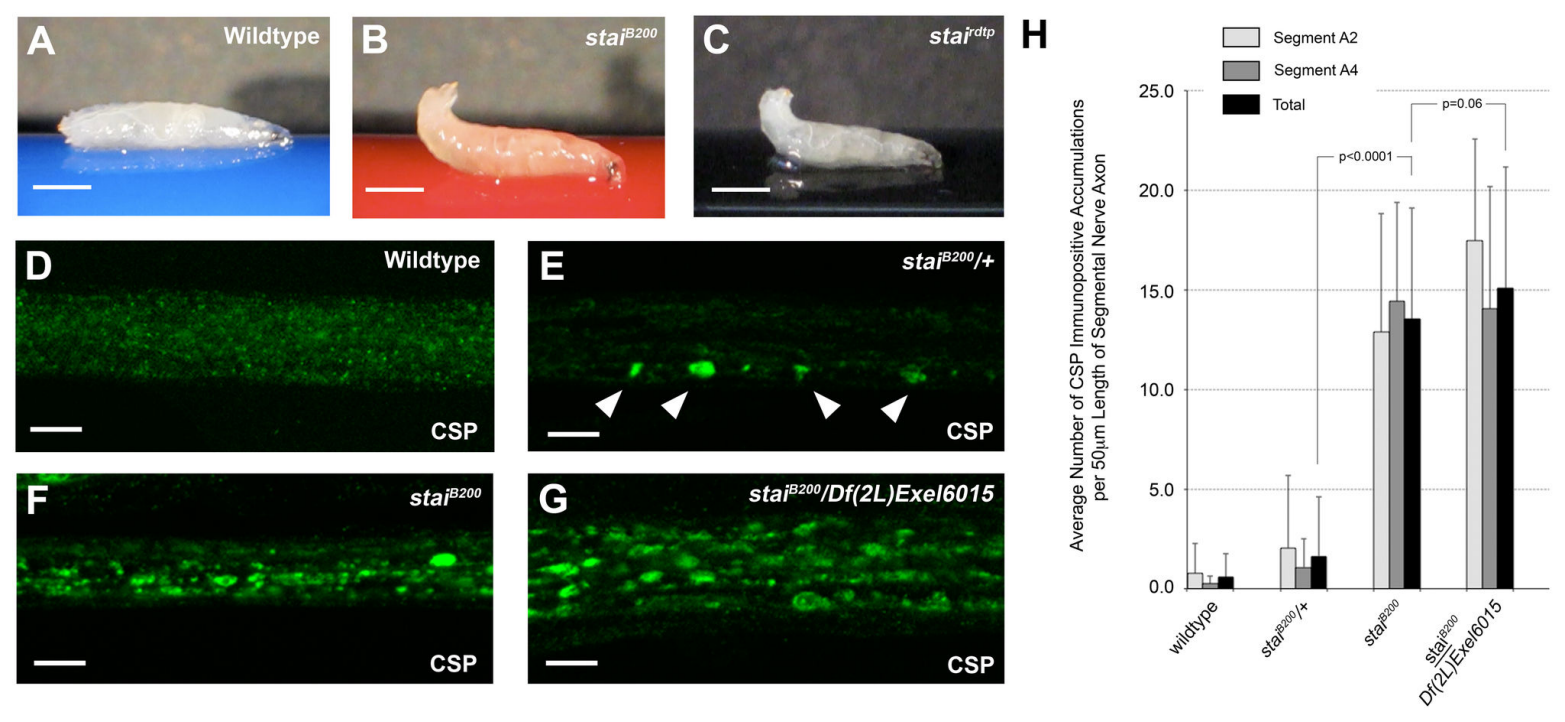
Loss of *stai* Function Results in Defects in Fast Axonal Transport. Mutations in *stai* result in phenotypes consistent with axonal transport defects. (**A**) Wildtype third instar larvae maintain a flat body posture when crawling. In contrast, homozygous *stai* ^*B200*^ larvae (**B**) and *stai* ^*rdtp*^ larvae (**C**) exhibit a posterior paralysis “tail flip” phenotype common for *Drosophila* mutants defective in axonal transport (Movies S1-S3). The tail-flip phenotype is also observed in heterozygous *stai* ^*B200*^/*stai* ^*rdtp*^ larvae (Movie S4). Confocal micrographs of sections of segmental nerve from wildtype (**D**), *stai* ^*B200*^ */+* heterozygous (**E**) and *stai* ^*B200*^ homozygous (**F**) third instar larvae stained with antibody against synaptic vesicle protein cysteine string protein (CSP). (**D**) The axons of wild type animals showed faint, punctate staining for CSP uniformly throughout the axons within the compound nerve bundle. (**E**) Although heterozygous *stai* ^*B200*^ larvae showed no obvious sluggishness or tail-flip phenotype when they crawled, mild accumulations of CSP were observed in some segmental nerve axons. (**F**) In contrast, homozygous mutant *stai* ^*B200*^ larvae exhibit abundant accumulations of CSP throughout the segmental nerve, confirming a disruption in axonal transport. (**G**) When *stai* ^*B200*^ is placed in heterozygous combination with *Df*(*2L*) *Exel6015*, a chromosomal deletion that spans the *stai* locus, there is an enhancement in the abundance of axonal accumulation of CSP. (**H**) The severity of axonal transport defects was quantified by averaging the number of accumulations of CSP immunopositive anterograde and retrograde membranous axonal cargos observed in the segmental nerve axons of *stai* deficient third instar larvae. To determine if there was an anterior-to-posterior gradient in the severity of axonal transport defects, regions of segmental nerve axons were analyzed as they passed through abdominal segment A2 (light grey bars) and abdominal segment A4 (dark grey bars). Data is also presented as total number of accumulations observed in both abdominal regions (black bars). The only significant regional difference observed in the abundance of axonal clogs in segmental nerve axons between abdominal segment A2 and A4 was in the axons examined from *stai* ^*B200*^ */Df*(*2L*) *Exel6015 larvae* (*p<0.0001*). Wild type larvae exhibited 0.57 ± 1.21 axonal clogs/50 µm segmental nerve axon in all axons examined. Homozygous *stai* ^*B200*^ larvae averaged 13.55 ± 5.56 axonal clogs/50 µm segmental nerve axon (p<0.0001). The average number of axonal clogs observed in the segmental nerve axons of *stai* ^*B200*^ */Df*(*2L*) *Exel6015* larvae was 15.10 ± 6.07 axonal clogs/50 µm segmental nerve axon. In panels A-C, the scale bar = 1mm. In panels D-G, the scale bar = 10 µm.

Homozygous *stai* ^*rdtp*^ and *stai* ^*B200*^ third instar larvae exhibited phenotypes characteristic of mutants with impaired axonal transport. Although *stai* mutant larvae were of comparable size to wild type larvae, their posterior regions were often weakly tapered and reduced in diameter as reported in other axonal transport mutants (data not shown) [48]. When homozygous *stai* ^*rdtp*^ and *stai* ^*B200*^ larvae crawled along the surface of a substrate, they exhibited a crawling behavior in which the posterior body segments sharply flipped upward after each peristaltic wave of muscle contraction during the crawling cycle (compare Figure 2A with 2B and 2C, Movies S1-S3). Heterozygous combination of *stai* ^*B200*^ with *stai* ^*rdtp*^ recapitulated the tail-flip phenotype (Movie S4). The larval posterior-paralysis or tail-flip phenotype is a hallmark of defective axonal transport in *Drosophila* and has been previously described for mutations in genes that encode components of MT motor protein complexes, such as kinesin and cytoplasmic dynein, as well as motor accessory proteins [28,29,33,35–37]. The phenotype is thought to result from a dorsal-ventral gradient in posterior muscle paralysis that causes an imbalance in contraction of the larval body wall muscles [29]. Interestingly, *stai* null third instar larvae also exhibited a posterior paralysis phenotype (gift of Pernille Rorth, IMCB, Singapore) (Movie S5).

We quantified the posterior paralysis and tail-flip phenotype of *stai* mutant larvae as described in the Materials and Methods. The posterior paralytic phenotype was incompletely penetrant, with 76.7% (n=92/120) of *stai* ^*B200*^ third instar larvae examined exhibiting a tail-flip (Table S1). Of the *stai* ^*B200*^ larvae exhibiting a posterior paralysis, 69.6% (n=64/92) had a robust tail-flip while 30.4% (n=28/92) had a mild tail-flip. The posterior paralysis phenotype was not observed in heterozygous *stai* ^*B200*^ */+* larvae (n=0/130) or larvae heterozygous for the chromosomal deletion *Df*(*2L*) *Exel6015* (n=0/120). Heterozygous combination of the *stai* ^*B200*^ allele with *Df*(*2L*) *Exel6015* increased the penetrance of third instar larvae exhibiting the posterior paralysis phenotype to 89.1% (n=90/101), with 75.6% (n=68/90) exhibiting a robust tail-flip and 24.4% (n=22/90) a mild tail-flip (Table S1). Thus, despite having almost undetectable levels of *stai* expression, as quantified by qRT-PCR (Figure 1C), the *stai* ^*B200*^ allele maintains some residual *stai* function and behaves like a strong hypomorphic mutation.

### *stai* Deficient Axons Exhibit Defects in Axonal Transport

Mutations in genes required for axonal transport not only cause a similar posterior paralysis and tail-flip phenotype in third instar larvae, but they also result in the accumulation of anterograde and retrograde membranous axonal cargo in the segmental nerves of these animals [29]. To determine if the posterior paralysis phenotype observed in *stai* mutant larvae resulted from impaired axonal transport, the distribution of cysteine string protein (CSP) in the axonal compartment was assayed by immunohistochemistry. CSP is a synaptic vesicle protein transported in a microtubule-dependent manner within the axons of larval segmental nerves [49,50]. In wildtype segmental nerve axons, CSP exhibits a uniform, punctate staining pattern throughout the axoplasm (Figure 2D). Heterozygous *stai* ^*B200*^ */+* larvae exhibited an average of 1.64 ± 2.95 (mean ± SD) CSP accumulations/50 µm segmental nerve axon examined (n=71 axon segments examined, p>0.05) (arrowheads Figure 2E, 2H). *stai* ^*B200*^ */+* larvae exhibit a normal crawling behavior and the absence of a posterior paralysis phenotype, indicating axonal transport is not significantly compromised in peripheral nerve axons. In contrast, homozygous *stai* ^*B200*^ third instar larvae exhibited 13.55 ± 5.56 aggregations of CSP/50 µm segmental nerve axon (n=52, p<0.0001, Figure 2F, 2H), consistent with an impairment in axonal transport. The CSP aggregations were not significantly enhanced in *stai* ^*B200*^ */Df*(*2L*) *Exel6015* larvae (15.10 ± 6.07 CSP accumulations/50 µm segmental nerve axon, n=57, p=0.06, Figure 2G, 2H). These results confirm that stai activity is required for efficient axonal transport in peripheral nerves.

To determine if there was an anterior to posterior gradient in the phenotypic severity of CSP aggregations in segmental nerve axons of *stai* mutant larvae, we quantified and compared the extent of axonal transport defects in regions of segmental nerve axons that passed through the more anterior abdominal segment A2 and the posterior abdominal segment A4 (Figure 2H). The only regional difference in the abundance of axonal clogs in segmental nerve axons was observed in axons from *stai* ^*B200*^ */Df*(*2L*) *Exel6015 larvae* (*p<0.0001*).

### Adult Viable *stai* Mutants Have Reduced Lifespans

**Figure 3:**
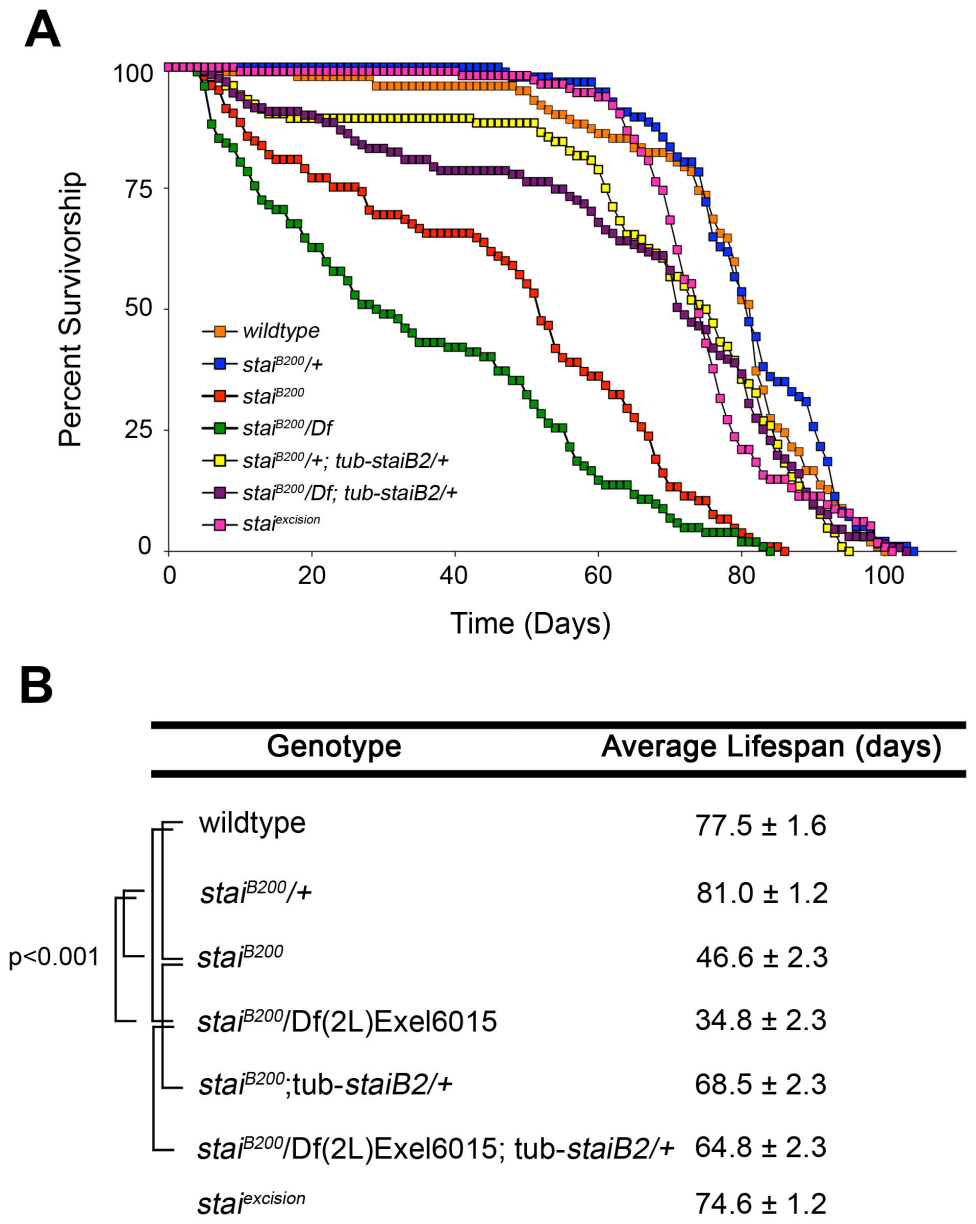
Loss of *stai* Function Reduces Adult *Drosophila* Lifespan. The lifespan of adult male *Drosophila* of seven different genotypes were measured at 25^o^C. A minimum of one hundred flies for each genotype were analyzed and the number of surviving flies were counted daily and survivorship curves generated (**A**). Adult wildtype *Drosophila* had an average lifespan of 77.5 ± 1.6 days (mean ± SE), not significantly different from the average lifespan of 81.0 ± 1.2 days observed in *stai* ^*B200*^ */+* heterozygous adults (p≥0.05) or the 80.2 ± 1.6 day average lifespan of *Df*(*2L*) *Exel6015/+* adults (p≥0.05) (data not shown). In contrast, the average lifespan of homozygous *stai* ^*B200*^ adults was 46.6 ± 2.3 days, significantly less than the average lifespan of wildtype, and heterozygous *stai* ^*B200*^/+ and *Df*(*2L*) *Exel6015/+* adults (p<0.001). Adult males of the genotype *stai* ^*B200*^/*Df*(*2L*) *Exel6015* had an even shorter average adult lifespan of 34.8 ± 2.3 days, significantly less than *stai* ^*B200*^ homozygous adult males (p<0.001). Introduction of an exogenous copy of a ubiquitously expressed transgene encoding the neuronal specific *staiB2* isoform, *tub-staiB2*, rescued the adult average lifespan of both *stai* ^*B200*^ homozygous adults to 68.5 ± 2.3 days (p<0.001) and *stai* ^*B200*^/*Df*(*2L*) *Exel6015* adults to 64.8 ± 2.3 days (p<0.001).

Our observations indicate that *Drosophila* can tolerate significant reductions in *stai* levels. Despite a large percentage of third instar larvae exhibiting a posterior paralysis due to impairment of axonal transport, a significant percentage of *stai* mutant animals manage to survive to the adult stage but exhibit significant reductions in average adult lifespan at 25^o^C (Figure 3). The *stai* ^*B200*^/+ heterozygote has an average adult lifespan of 81.0 ± 1.2 days, not significantly different from the 77.5 ± 1.6 days observed in the wildtype (P≥0.05) (Figure 3A, B) or the 80.2 ± 1.6 days in *Df*(*2L*) *Exel6015/+* adults (P≥0.05) (data not shown). In contrast, a significant reduction in average adult lifespan was observed for *stai* ^*B200*^ adults, averaging 46.6 ± 2.3 days (P<0.0001) (Figure 3A, B). The average adult lifespan was reduced even further, to 34.8 ± 2.3 days (P<0.0001) in *stai* ^*B200*^/*Df*(*2L*) *Exel6015* adult males (Figure 3A, 3B).

### Adult Viable *stai* Mutants Exhibit a Progressive Bang-Sensitive Seizure Phenotype

While performing the lifespan analysis on adult viable *stai* mutants, we observed they exhibit a temporary paralysis in response to the mechanical activity of being transferred to new food vials. This phenotype is similar to that observed in the "bang-sensitive" class of genes, mutant alleles of which cause neurological defects characterized by a paralysis or seizure in response to mechanical stimulation. Mutant alleles of a number of genes have been identified that exhibit a bang-sensitive phenotype including *bang-sensitive* (*bas*)*, bang-senseless* (*bss*)*, technical knockout* (*tko*)*, slam dance* (*sda*) and *easily shocked* (*eas*). These genes are involved in diverse cellular processes including ion transport, Ca^2+^ release and mitochondrial metabolism [51,52]. Typically, the seizures become easier to trigger, and the subsequent paralysis increases in duration, with increasing age [53].

We quantified the response of *stai* mutant animals to mechanical stimulation by performing a bang-sensitive assay on adult male flies of different ages. We observed a normal response to, and recovery from, mechanical stimulation for all genotypes tested at 1, 7 and 14 days post eclosion (Tables S2 and S3). However, 20.9% of *stai* ^*B200*^ males tested at 21 days post eclosion (n=67) exhibited bang-sensitive paralysis with a mean recovery period of 48.6 ± 26.7 seconds (mean ± SD). During the bang-sensitive paralysis, flies laid motionless on the media at the bottom of the vial for periods ranging from 10 seconds to 3 minutes, depending on age. Upon recovery, flies exhibited a hyperactive phase, known as a recovery seizure, in which they vigorously flapped their wings and moved their legs for approximately 4-6 seconds, prior to righting themselves to a standing position and walking away. By 42 days, 75.8% of *stai* ^*B200*^ adults exhibited bang-sensitive paralysis, phenotype with an increase in the mean recovery period to 76.6 ± 41.4 seconds (n=62) (Movie S9). By 56 days, 91.7% of *stai* ^*B200*^ adults exhibited the bang-sensitive response with a mean recovery period of 145.4 ± 63.8 seconds (n=144).

Interestingly, the progressive bang-sensitive phenotype was also observed in heterozygous *stai* ^*B200*^/+ and *stai* ^*rdtp*^/+ adults, but with a delayed onset and a marked reduction in the recovery period following paralysis compared to *stai* ^*B200*^ adults (Tables S2 and S3). A bang-sensitive response to mechanical stimulation was first observed in heterozygous *stai* mutants at 42 days post eclosion where 25.2% of *stai* ^*B200*^/+ (n=111) and 18.0% of *stai* ^*rdtp*^/+ adults (n=50) exhibited the phenotype. The mean recovery period for *stai* ^*B200*^/+ adults was 55.7 ± 27.4 seconds and for *stai* ^*rdtp*^/+ adults was 55.4 ± 43.7 seconds. By 56 days post eclosion, the penetrance of the bang-sensitive phenotype had increased to 58.0% of *stai* ^*B200*^ */+* and 52.9% of *stai* ^*rdtp*^/+ adults, with an increase in the average recovery period to 85.6 ± 43.6 seconds and 124.4 ± 71.8 seconds respectively (Tables S2 and S3). We conclude that viable adult *stai* mutants exhibit a progressive, age-dependent bang-sensitive phenotype to mechanical stimulation, reflective of compromised nervous system function.

### Reversion and Rescue of *stai* Mutant Phenotypes

**Figure 4:**
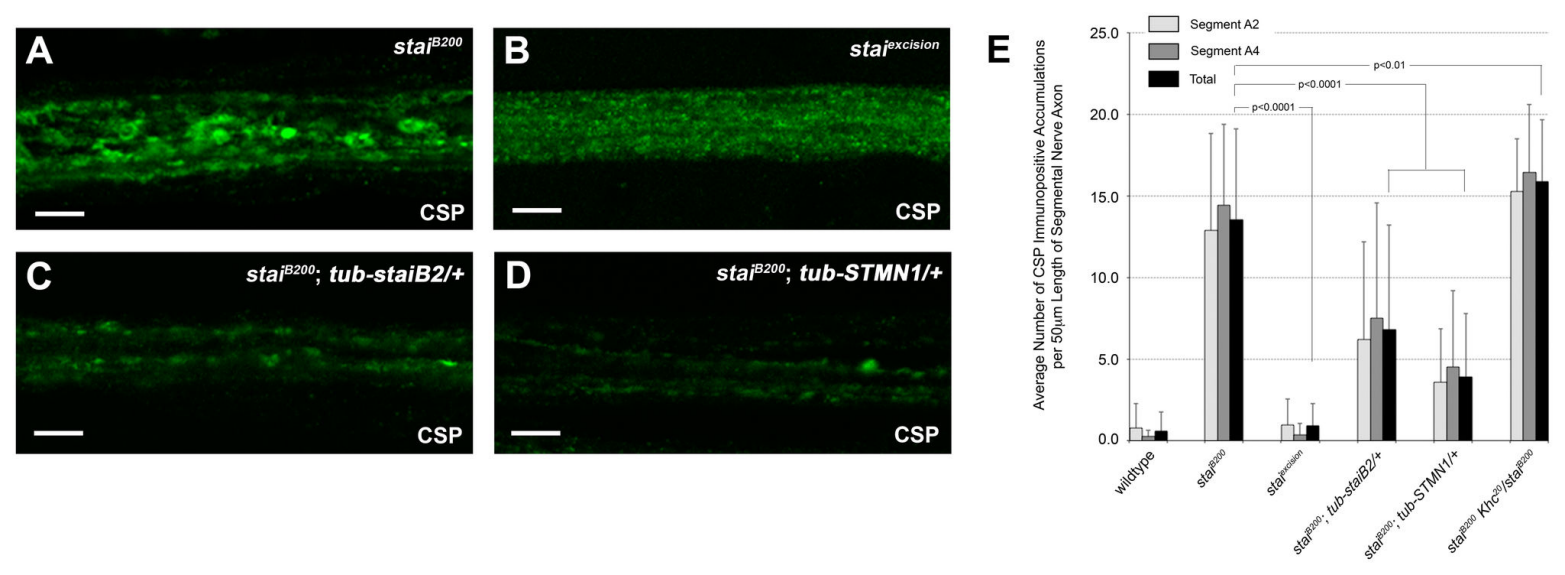
Reversion and Genetic Rescue of Fast Axonal Transport Defects in *stai* Deficient Axons. Confocal micrographs of segmental nerves from (**A**) *stai* ^*B200*^ homozygous third instar larvae stained with antibodies against synaptic vesicle protein cysteine string protein (CSP). Note the abundant accumulation of CSP throughout the segmental nerve, indicative of a disruption in fast axonal transport. (**B**) In contrast, precise excision of the mutagenic *piggyBac* element *PBac*{*5HPw* ^*+*^}, responsible for the loss of *stai* function in *stai* ^*B200*^, completely restores axonal transport and reverts the CSP staining pattern in the axon to the punctate, uniform distribution observed in wildtype axons (compare Figure 4B with Figure 2D) (Movie S6). (**C**) Introduction of a single copy of a ubiquitously expressed transgene encoding the neuronal specific *staiB2* isoform, *tub-staiB2*, ameliorated the accumulation of CSP observed in the segmental nerves of *stai* deficient axons of *stai* ^*B200*^ larvae. (**D**) Introduction of a single copy of a ubiquitously expressed human transgene encoding the *stai* gene, *tub-STMN1*, also diminishes the accumulation of CSP in the segmental nerves of *stai* deficient axons of *stai* ^*B200*^ larvae, indicating the *Drosophila* and human stai proteins are functional orthologs. (**E**) The severity of axonal transport defects was quantified by averaging the number of accumulations of CSP immunopositive anterograde and retrograde membranous axonal cargos observed in the segmental nerve axons of *stai* deficient third instar larvae. Axonal transport defects are reverted to wildtype in the axons of *stai* ^*excision*^ larvae that had 0.91 ± 1.37 CSP accumulations/50 µm segmental nerve axon (p<0.0001). Axonal transport defects are reduced in the segmental nerve axons of stai^B200^ larvae expressing a single copy of the *Drosophila* rescue transgene *tub-staiB2*, 6.79 ± 6.43 axonal clogs/50 µm segmental nerve axon (p<0.0001), and the human transgene *tub-STMN1*, 3.92 ± 3.87 axonal clogs/50 µm segmental nerve axon (p<0.0001). Results are reported as the average number of axonal clogs (mean ± SD) per 50 µm segmental nerve axon. A small but significant increase in the severity of axonal clogs was observed in the segmental nerve axons of *stai* ^*B200*^ *Khc* ^20^/*stai* ^*B200*^ larvae (15.89 ± 3.80 CSP accumulations/50 µm segmental nerve axon, p<0.01) compared to *stai* ^*B200*^ third instar larvae. In panels A-D, the scale bar = 10 µm.

To demonstrate that all observed phenotypes are due to reduction in *stai* activity, we mobilized the *piggyBac* element in the *stai* ^*B200*^ allele to generate the precise excision allele *stai* ^*excision*^ that restored the *stai* gene to its original structure and acted as a genotype matched control. All *stai* ^*B200*^ mutant phenotypes were reverted to wildtype in *stai* ^*excision*^ animals. Homozygous *stai* ^*excision*^ third instar larvae did not exhibit the posterior paralysis and tail-flip phenotype observed in *stai* ^*B200*^ larvae (n=0/120) (Table S1 and Movie S6). In addition, the aggregation of CSP immunoreactive vesicles in larval segmental nerves of *stai* ^*excision*^ larvae was not significantly different than wildtype (0.91 ± 1.37 CSP accumulations/50 µm segmental nerve axon, n=70, p=0.68, compare Figure 4A and B Figure 4E). The average adult lifespan was also reverted to wildtype (74.6 ± 1.2 days) (P≥0.05) (Figure 3A, 3B). Finally, *stai* ^*excision*^ adults did not exhibit the progressive bang-sensitive phenotype observed in *stai* mutant adults (Tables S2 and S3).

Further, we were able to partially rescue all phenotypes observed in *stai* ^*B200*^ third instar larvae and viable adults with the ubiquitous ectopic expression of *staiB2*, a neuronal specific *Drosophila* *stai* isoform (*tub-staiB2* - gift of Pernille Rorth, IMCB, Singapore) [54]. The severity of the posterior paralysis phenotype was greatly reduced in *stai* ^*B200*^ *; tub-staiB2/+* larvae compared to *stai* ^*B200*^ larvae (Table S1 and Movie S7). Immunohistological analysis revealed that the *tub-staiB2* transgene significantly reduced the severity of the aberrant accumulation of CSP observed in segmental nerves of homozygous *stai* ^*B200*^ larvae (6.79 ± 6.43 CSP accumulations/50 µm segmental nerve axon, n=52, p<0.0001, compare Figure 4A and 4C Figure 4E). The *tub-staiB2* transgene also delayed the onset and ameliorated the severity of the bang-sensitive phenotype exhibited by *stai* ^*B200*^ males from 75.8% to 5.8% (n=52) at 42 days post eclosion, while significantly reducing the mean recovery period to 39.7 ± 12.5 seconds from 76.6 ± 41.4 seconds (Tables S2 and S3). A single copy of the *tub*-*staiB2* transgene also significantly increased average adult lifespan to 68.5 ± 2.3 days in *stai* ^*B200*^; *tub-staiB2*/+ adults, and to 64.8 ± 2.3 days in *stai* ^*B200*^/*Df*(*2L*) *Exel6015*; *tub-staiB2*/+ adults compared to the average lifespan of *stai* ^*B200*^ and *stai* ^*B200*^ */Df*(*2L*) *Exel6015* adults (P<0.001) (Figure 3B). While the ectopic expression of *staiB2* rescued the posterior paralysis and aberrant accumulation of CSP in segmental nerve axons of *stai* ^*rdtp*^ homozygous larvae (data not shown), it did not rescue lethality, supporting the idea that the *stai* ^*rdtp*^ chromosome carries a linked, lethal mutation.

Finally, we were also able to rescue the *stai* mutant phenotypes with the ubiquitous ectopic expression of the human stathmin gene, *STMN1*. A single copy of the *tub-STMN1* transgene significantly reduced the aberrant accumulation of CSP in the segmental nerve axons of *stai* ^*B200*^ larvae (3.92 ± 3.87 CSP accumulations/50 µm segmental nerve axon, n=84, p<0.0001, compare Figure 4A and 4D Figure 4E). The *tub-STMN1* transgene also reduced the percentage of *stai* ^*B200*^ larvae that exhibited a posterior paralysis phenotype from 76.7% (n=92/120) to 39.6% (n=44/111) (Table S1). In addition, the severity of the posterior paralysis phenotype was reduced to only 9.1% (n=4/44) of *stai* ^*B200*^ *; tub-STMN1* larvae that exhibited a robust tail-flip from 69.6% (n=64/92) of *stai* ^*B200*^ larvae (Table S1 Movie S8). These data support previous observations that the *Drosophila* and human stathmin proteins are functionally equivalent orthologs.

Although all observed *stai* mutant phenotypes could be rescued with both *Drosophila staiB2* and human *STMN1* transgenes, rescue was incomplete. This was likely due to the fact that the rescuing *Drosophila staiB2* transgene expresses only one of the four known stai protein isoforms, which may have been insufficient for complete rescue. In addition, expression of both transgenes was under the control of the tubulin promoter that may not have provided the correct spatial and temporal expression of the transgenes.

### Loss of *stai* Function Reduces Tubulin and Kinesin Heavy Chain Protein Levels

**Figure 5:**
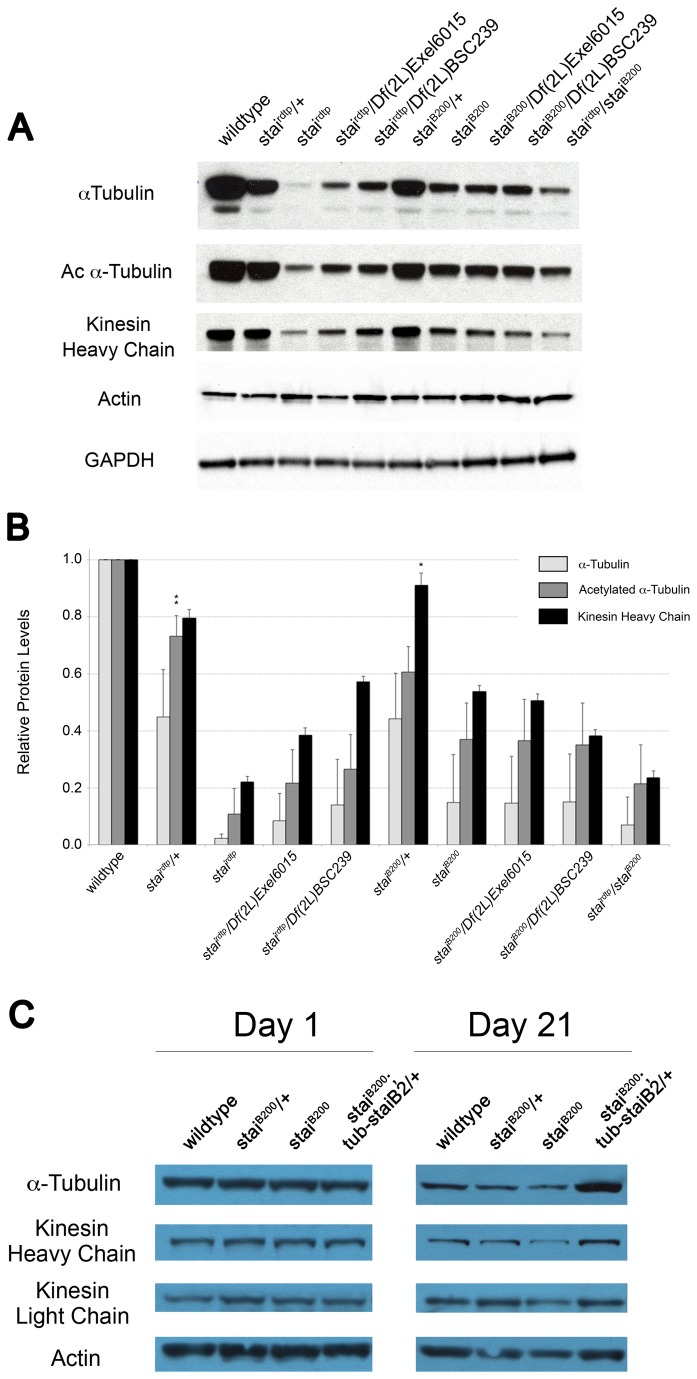
*stai* Mutants Have Reduced Levels of α-Tubulin, Acetylated α-Tubulin, and Kinesin Heavy Chain Protein. (**A**) Representative western blots of total protein extracted from *stai* mutant third instar larvae. Heterozygous *stai/+* mutant larvae exhibit mild reductions in the levels of α-tubulin and acetylated α-tubulin compared with wild type larvae. Homozygous *stai* mutant larvae have dramatic reductions in the levels of α-tubulin, acetylated α-tubulin and the heavy chain subunit of the microtubule motor protein kinesin compared to wildtype. *Df*(*2L*) *BSC239* represents a second chromosomal deficiency that excludes the *stai* gene. GAPDH is used as a loading control. (**B**) Quantification of western blots of total protein extracts isolated from third instar larvae probed with antibodies against α-tubulin (mAB DM1A), acetylated α-tubulin (mAB 611B1), and kinesin heavy chain (AKIN01). Quantification was performed as described in the Materials and Methods. Results are normalized against wildtype and presented as mean ± S.D. Statistical comparison reveals that the levels of α-tubulin, acetylated α-tubulin, and kinesin heavy chain proteins for all genotypes examined differ significantly from wildtype (p<0.001), with the exception of levels of acetylated α-tubulin in *stai* ^*rdtp*^ */+* larvae (**p<0.01) and kinesin heavy chain in *stai* ^*B200*^/+ (*p<0.05). (**C**) Western blot analysis of total protein extracted from *stai* mutant adult *Drosophila* aged 1 day and 21 days post eclosion. The levels of α-tubulin, and conventional kinesin motor heavy and light chain subunits are present at equal levels in wildtype, *stai* ^*B200*^ */+, stai* ^*B200*^ and *stai* ^*B200*^; *tub-staiB2* adults 1 day post eclosion. At 21 days post eclosion, the age we observe *stai* ^*B200*^ adults exhibiting a bang-sensitive response to mechanical stimulation, the levels of α-tubulin, and conventional kinesin motor heavy and light chain subunits are noticeably reduced. Introduction of an exogenous copy of the rescuing transgene *tub-staiB2*, not only increases the levels of α-tubulin, but also increases levels of the conventional kinesin motor heavy and light chain subunits.

Since stai functions as a tubulin-binding protein and is known to regulate MT dynamics, we used western blot analysis to examine and quantify tubulin levels in total protein extracts isolated from *stai* mutant third instar larvae and adults. Levels of α-tubulin were significantly reduced in *stai* ^*B200*^/+ larvae compared with wild type controls (p<0.0001, Figure 5A, 5B). A more pronounced reduction in the levels of α-tubulin protein was observed in homozygous *stai* ^*B200*^ larvae (Figure 5A). Third instar *stai* ^*B200*^ larvae had levels of α-tubulin that were 15.0 ± 16.6% (mean ± SD) of that observed in wild type larvae (p<0.0001, Figure 5A, 5B). In order to assess the stability of polymerized MTs, we also assayed for levels of acetylated α-tubulin in *stai* mutant larvae. The levels of acetylated α-tubulin were also significantly reduced in *stai* ^*B200*^ */+* mutant larvae (p<0.0001, Figure 5A, 5B), and more greatly reduced in homozygous stai^B200^ mutant larvae to 36.9 ± 12.9% of the levels observed in wildtype (p<0.0001, Figure 5A, 5B). The posterior paralysis displayed by *stai* homozygotes is reminiscent of the ‘tail flip’ phenotype first described in Kinesin heavy chain (Khc) mutant larvae [29]. We therefore examined protein extracts from *stai* mutants to see if Khc levels were affected. We observed a significant reduction in the level of Khc, the force generating subunit of the MT motor protein kinesin-1, in *stai* mutant larvae (Figure 5A). Heterozygous *stai* ^*B200*^ */+* larvae had levels of Khc protein that were 91.2 ± 4.2% of that observed in wildtype (p<0.05) whereas *stai* ^*B200*^ larvae had levels that were 53.8 ± 2.3% of wildtype (p<0.0001, Figure 5A, 5B). Collectively, these data suggest a general impairment of the MT-based transport system following loss of *stai* function in *Drosophila* .

Given the observed effect of loss of *stai* function on the levels of α-tubulin, acetylated α-tubulin and Khc protein in third instar larvae (Figure 5A), and the observation that ectopic expression of *staiB2* rescued the shortened lifespan of *stai* ^*B200*^ adults (Figure 3A), we analyzed the levels of α-tubulin and kinesin-1 motor components in adult *stai* mutant animals at 1 day and 21 days post-eclosion (Figure 5C). These ages were chosen because they corresponded to the age at which a bang-sensitive response to mechanical stimulation was first observed in *stai* deficient animals (Table S2). At 1 day post-eclosion, the levels of α-tubulin, Khc and Kinesin light chain (Klc) subunits were equivalent across all genotypes analyzed (Figure 5C). However, by 21 days post-eclosion, there was a marked reduction in the levels of α-tubulin, Khc and Klc proteins in *stai* ^*B200*^ adults. Surprisingly, ectopic expression of *staiB2* not only restored the levels of α-tubulin in *stai* ^*B200*^ *; tub-staiB2/+* adults as predicted, but also increased the levels of both the Khc and Klc protein subunits (Figure 5C).

### Genetic Reductions in the Kinesin Heavy Chain Gene Enhance *stai* Phenotypes

Because loss of *stai* function resulted in decreased levels of Khc protein in third instar larvae, and ectopic expression of *staiB2* restored reduced levels of conventional kinesin motor protein subunits in adult flies, we assayed for a genetic interaction between *stai* and *kinesin* by combining a genetic reduction in the gene that encodes the conventional kinesin heavy chain protein, *Khc*, with mutations in the *stai* gene. Loss of a single copy of *Khc* did not cause a posterior paralysis phenotype or enhance the axonal transport defects observed in *stai* ^*B200*^/+ larvae (data not shown). However, introduction of the *Khc* ^20^ null allele into the homozygous *stai* ^*B200*^ genetic background increased the penetrance of the posterior paralysis phenotype observed in *stai* ^*B200*^ *Khc* ^20^/*stai* ^*B200*^ third instar larvae to 97.1% (n=101/104) compared with only 76.7% (n=92/120) of *stai* ^*B200*^ homozygous larvae (Table S1). In addition, we observed a small, but significant, increase in the severity of aberrant accumulation of CSP in the segmental nerve axons of *stai* ^*B200*^ *Khc* ^20^/*stai* ^*B200*^ compared to *stai* ^*B200*^ third instar larvae (15.89 ± 3.80 CSP accumulations/50 µm segmental nerve axon, n=48, p<0.01, Figure 4E).

### Loss of *stai* Function Alters the Integrity of Axonal MTs

MTs are the main cytoskeletal component of axons. The motor proteins kinesin and cytoplasmic dynein mediate organelle transport on MT tracks within the axon. Given the known function of stai as a regulator of MT dynamics and our demonstration that loss of *stai* function disrupts axonal transport and reduces the levels of α-tubulin and acetylated α-tubulin in third instar larvae, we assayed the integrity of MTs in segmental nerve axons by immunostaining *stai* mutant larvae with antibody against the neuronal specific MAP Futsch (mAb 22c10), the *Drosophila* homolog of the vertebrate MAP1B protein that reveals stabilized MTs in neurons [55].

**Figure 6:**
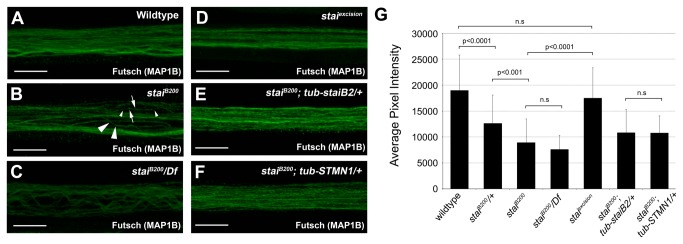
Loss of *stai* Function Reduces Futsch (MAP1B) Protein Levels in Axons and Alters the Integrity of Axonal Microtubules. (**A**–**F**) Confocal micrographs of segmental nerve axons from third instar larvae immunostained with antibody against the MAP Futsch (mAb 22c10). (**A**) Wild type larvae have well defined Futsch staining in individual axons within segmental nerve bundles. (**B**) In contrast, loss of *stai* function results in an inconsistent distribution of Futsch protein along the length of individual axons within segmental nerve bundles. In some regions of an axon, there is prominent Futsch staining (arrows) whereas Futsch staining is noticeably reduced in other areas of the same axon (small arrowheads). In addition, loss of *stai* function causes gross perturbations in axon morphology (large arrowheads). (**C**) The perturbations in axon morphology are more profound in segmental nerve axons from *stai* ^*B200*^ */Df*(*2L*) *Exel6015* larvae. (**D**) Precise excision of the *piggyBac* element *PBac*{*5HPw^+^*}^*B200*^ reverts the observed phenotypes to wildtype. The reduction in Futsch staining intensity and perturbations in axon morphology observed in *stai* deficient segmental nerve axons can be ameliorated with the ubiquitous ectopic expression of *Drosophila staiB2* (**E**) or human STMN1 (**F**). (**G**) Quantification of average pixel intensity of Futsch immunostained axons of third instar larval segmental nerves. Results are presented in arbitrary units (a.u.) of fluorescence intensity (mean ± S.D.). In panels A-F the scale bar = 10 µm.

Wild type larvae have a well-defined MT network in segmental nerve axons, as determined by the even distribution of Futsch protein along the length of individual axons within the compound nerve (Figure 6A). In contrast, the distribution of Futsch protein appeared to be disrupted and less consistent along the length of segmental nerve axons of *stai* mutant larvae, intense and prominent in some axonal regions (arrows Figure 6B), yet noticeably reduced and absent in other regions of the same axon (small arrowheads Figure 6B). We interpret this observation as a disruption in Futsch-stabilized MTs within *stai* deficient segmental nerve axons.

We also observed a dosage dependent reduction in the intensity of Futsch staining in the axons of segmental nerve bundles of *stai* deficient larvae. We quantified and compared the average pixel intensity of Futsch staining in axons of third instar larvae segmental nerves (Table S4). The axons of *stai* ^*B200*^/+ third instar larvae exhibited a significant reduction in the average pixel intensity of Futsch immunostained segmental nerve axons compared to that observed in wildtype axons (WT 19000 ± 5200 arbitrary units (a.u.), n=43; *stai* ^*B200*^/+ 12600 ± 4800 a.u, n=42, p<0.0001, Figure 6G, Table S4). The average intensity of Futsch staining was further reduced in the axons of *stai* ^*B200*^ homozygous third instar larvae (9000 ± 3000 a.u, n=32 p<0.0001, compare Figure 6A and B) and greater yet in the axons of *stai* ^*B200*^/*Df*(*2L*) *Exel6015* larvae (7600 ± 2200 a.u, n=30, p<0.0001, compare Figure 6A and C) compared to wildtype.

In addition to acting as tracks to support transport through the axon, MTs also provide internal structural support that influences and maintains the morphology of the axon. Individual axons that comprise the compound segmental nerve of wild type larvae are arranged in linear, ordered bundles (Figure 6A). In contrast, *stai* deficient larvae exhibited alterations in the morphology of individual axons within the segmental nerve that are not observed in wildtype or *stai/+* larvae (large arrowheads Figure 6B). Individual axons were often bent and non-linear in *stai* deficient segmental nerves. This observed alteration in axon morphology was more pronounced and affects a greater number of segmental nerve axons of *stai* ^*B200*^ */Df*(*2L*) *Exel6015* larvae (Figure 6C).

**Figure 7:**
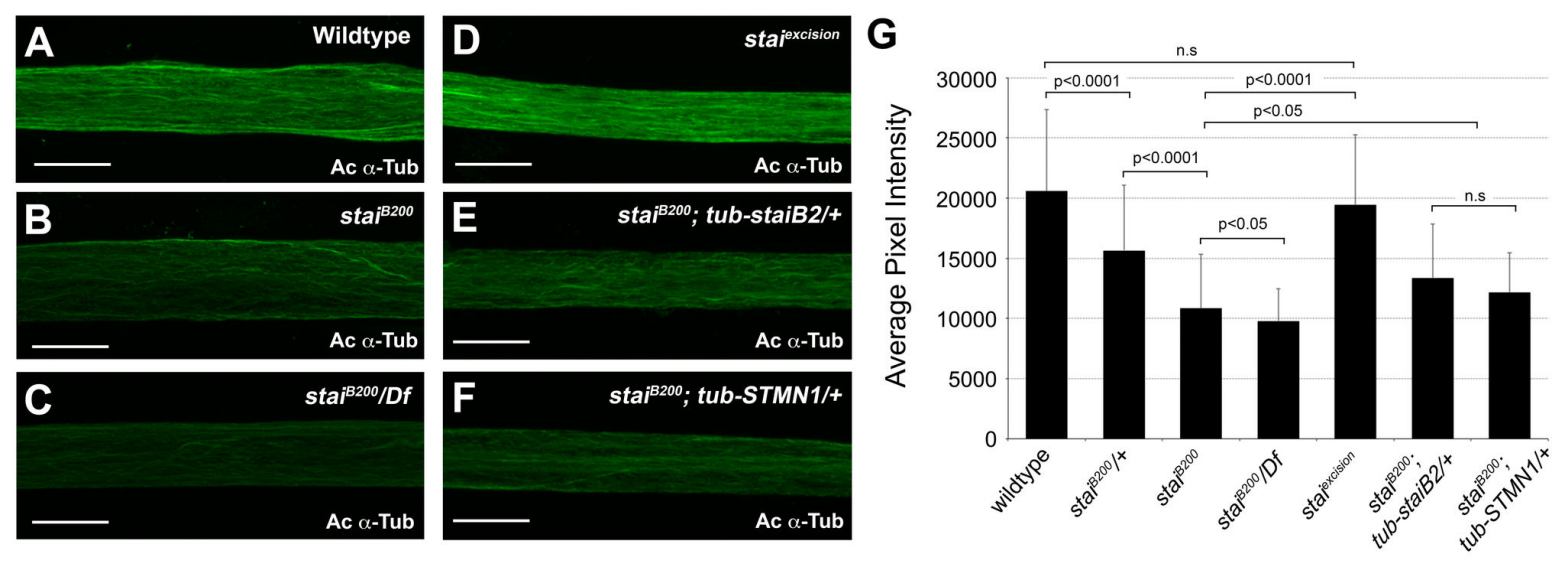
Loss of *stai* Function Reduces Levels of Stabilized Axonal Microtubules. (**A**–**F**) Confocal micrographs of segmental nerve axons from third instar larvae immunostained with antibody against acetylated α-tubulin (mAb 611B1). Axons from (**A**) wildtype, (**B**) *stai* ^*B200*^ and (**C**) *stai* ^*B200*^ */Df*(*2L*) *Exel6015* larvae. (**D**) Precise excision of the *piggyBac* element *PBac*{*5HPw^+^*}^*B200*^ reverts the reduction in acetylated α-tubulin back to wild type levels. The reduction in acetylated α-tubulin staining intensity observed in *stai* deficient segmental nerve axons is significantly restored with the ubiquitous ectopic expression of *Drosophila staiB2* (**E**) or human STMN1 (**F**). (**G**) Quantification of average pixel intensity of acetylated α-tubulin immunostained axons of third instar larval segmental nerves. Results are presented in arbitrary units (a.u.) of fluorescence intensity (mean ± S.D.). In panels A-F the scale bar = 10 µm.

We also assessed the integrity of axonal MTs in segmental nerve axons directly by immunostaining *stai* mutant larvae with antibody against acetylated α-tubulin, indicative of stable microtubules [56]. The axons of *stai* ^*B200*^ */+* third instar larvae exhibited a significant reduction in the average pixel intensity of acetylated α-tubulin immunostained segmental nerves compared to the axons of wild type larvae (WT 20600 ± 6800 a.u., n=70; *stai* ^*B200*^ */+* 15700 ± 5400, n=110, p<0.0001, Table S5). The average pixel intensity of acetylated α-tubulin staining was reduced further in the axons of *stai* ^*B200*^ larvae (10800 ± 4500 a.u., n=230, p<0.0001, compare Figure 7A and B, Table S5) and even further in *stai* ^*B200*^ */Df*(*2L*) *Exel6015* axons (9800 ± 2700 a.u., n=125, p<0.05, Figure 7C, Table S5). Thus, loss of *stai* function results in a reduction in the stabilized microtubule network of peripheral nerve axons in a dosage dependent manner.

The ubiquitous, exogenous expression of *Drosophila staiB2* or human STMN1 rescued the altered morphology of individual axons within segmental nerve bundles of *stai* ^*B200*^ larvae (compare Figure 6C with Figure 6E, F). The rescue transgenes improved, but did not significantly increase, the average pixel intensity of Futsch staining detected in the axons of *stai* ^*B200*^ *; tub-staiB2/+* (10800 ± 4100 a.u., n=24, p=0.12) and *stai* ^*B200*^ *; tub-STMN1/+* (10800 ± 1800 a.u., n=22, p=0.14) larvae compared with *stai* ^*B200*^ homozygous larvae (9000 ± 3000 a.u., n=32). However, the rescue transgenes did significantly increase the average pixel intensity of acetylated α-tubulin staining detected in the axons of both *stai* ^*B200*^ *; tub-staiB2/+* (13400 ± 4500 a.u., n=80, p<0.0001, compare Figure 7A and E) and *stai* ^*B200*^ *; tub-STMN1/+* (12200 ± 3300, n=115, p<0.05, compare Figure 7A and F) third instar larvae.

### Loss of *stai* Function Alters the Integrity of Muscle MTs

The observed phenotypes following loss of *stai* function are consistent with a reduction in the level of polymerized MTs in the axons of *stai* mutant animals, contradictory to the function of stai as a MT depolymerizing protein. Because the integrity of MTs within individual segmental nerve axons is difficult to assess by direct visualization, we also looked at the effect of loss of *stai* function on the architecture of the MT network in body wall muscle 6 from abdominal segment A4 of our *stai* mutant larvae.

The MT network in muscle cells from wild type larvae is very well defined, enriched around the nucleus and extending into the cytoplasm (Figure S1 A). The MT network of *stai/+* muscle cells appears indistinguishable from wildtype (data not shown). The MT network is noticeably less dense, however, both around the nucleus and within the cytoplasm of muscle cells from *stai* ^*B200*^ mutant larvae (compare Figure S1 A and B). The reduction in polymerized MTs is even more pronounced in *stai* ^*B200*^ */Df*(*2L*) *Exel6015* larvae (compare Figure S1 A, B and C) confirming that the *stai* ^*B200*^ allele retains residual *stai* activity. This observation directly supports our results from western blot data regarding the observed reduction in levels of acetylated α-tubulin in *stai* and *stai/Df*(*2L*) *Exel6015* third instar larvae. Interestingly, we detect an abundance of fragmented MTs within the cytoplasm of *stai* deficient cells (arrowheads Figure S1 B, C).

We quantified the density of perinuclear MTs in muscle cells of our *stai* mutant animals and found that MT density was significantly reduced in *stai* ^*B200*^ (P<0.05) and *stai* ^*B200*^ */Df*(*2L*) *Exel6015* (P<0.01) larvae (Figure S1 G). The architecture of the MT network was restored to wildtype in *stai* ^*excision*^ muscle cells (Figure S1 D) and rescued with expression of both the *tub-staiB2* (Figure S1 E), and the human *tub-STMN1* transgenes (Figure S1 F). Rescue of the MT network in *stai* ^*B200*^ larvae with the *tub-staiB2* transgene resulted in a perinuclear MT density greater than wildtype (P<0.01) (Figure S1 G).

## Discussion

The development and function of the nervous system is dependent on a highly dynamic, yet tightly regulated, microtubule (MT) cytoskeleton (reviewed in [2,57]). The stathmin (*stai*) family of proteins comprise a group of MT destabilizing proteins that indirectly function to regulate microtubule dynamics by binding and sequestering free tubulin and stimulating MT depolymerization [16]. It has been previously demonstrated in *Drosophila* that *stai* is maternally contributed to the oocyte and is required for the development of the embryonic nervous system [58]. The recessive, hypomorphic alleles we have isolated in the *Drosophila* *stai* gene have allowed us to circumvent the requirement for *stai* during embryonic development, and identify a function in the maintenance of axonal MTs in the mature nervous system, necessary to support and sustain axonal transport.

Collectively, our results indicate that *stai* function is required for axonal transport. Phenotypes consistent with impaired axonal transport, including a posterior paralysis tail-flip and accumulation of synaptic proteins in peripheral segmental nerve axons, were observed in *stai* mutant larvae. Furthermore, genetic perturbations that independently affect axonal transport (such as a 50% reduction in Kinesin heavy chain (Khc) copy number) enhance the severity of the stai mutant phenotype including the accumulation of synaptic proteins in segmental nerve axons. We also observe reductions in the levels of tubulin, acetylated tubulin and kinesin heavy and light chain protein subunits in our *stai* mutants. Importantly, Khc levels were reduced by less than 50%. Since *Khc* hemizygotes are phenotypically normal, our data argue that the observed reduction in Khc levels contribute to the *stai* mutant phenotype, but are not its primary cause. These phenotypes are ameliorated by ectopic expression of a *Drosophila* *stai* transgene encoding a neuronal specific *stai* isoform. In addition, we are able to rescue the observed phenotypes with ectopic expression of the human gene *STMN1*. Mutations in cytoplasmic dynein subunits and motor protein accessory factors also result in axonal transport defects. A potential direction for future studies could be to comprehensively examine how microtubule motor protein expression is affected in *Drosophila* *stai* mutants. A comparison of *STMN1* ^*+/+*^ vs. *STMN1* ^*-/-*^ mouse embryonic fibroblasts identified motor proteins as the largest group of genes with altered expression profiles. Interestingly, while differential expression of 13 kinesin motor subunits was detected alteration in dynein motor subunit expression was not observed, and only 5 genes encoding dynein light or intermediate chains showed altered expression [59].

The severity of axonal transport blockages observed in *stai* mutant axons is significantly less than that observed in microtubule motor mutants. It has previously been reported that *Drosophila* third instar larvae null for the microtubule motor proteins *Dynein heavy chain* (*Dhc*) and *Khc* exhibit 250 ± 25 clogs per 50 µm length of segmental nerve axon [60]. Third instar larvae of the genotype *stai* ^*B200*^/*Df*(*2L*) *Exel6015* exhibit on average 15.10 ± 6.07 clogs per 50 µm length of segmental nerve, significantly less than reported in *Dhc* and *Khc* null third instar larvae. The severity of axonal transport blockages observed in *stai* mutant larvae, however, is equivalent to levels observed in larvae null for the *amyloid precursor protein-like* gene *Appl* [61]. Interestingly, *Appl* null larvae are also homozygous viable, suggesting that the severity of the disruption of axonal transport may determine viability and in part explain why a significant percentage of *stai* ^*B200*^ and *stai* ^*B200*^/*Df*(*2L*) *Exel6015* mutants are homozygous viable.

We routinely recover viable *stai* ^*B200*^ */Df*(*2L*) *Exel6015* adult flies, despite undetectable levels of *stai* transcript as measured by qPCR analysis, suggesting that *stai* function is not necessary for viability. This observation is consistent with a recent report that *stai* knockout flies are viable, indicating that *stai* is a non-essential gene [45]. The *stai* mutant adult flies exhibit a significantly reduced lifespan and a progressive, age-dependent epileptic-like seizure and paralysis in response to mechanical stimulation. To our knowledge, the bang-sensitive phenotype has not been previously reported in motor-protein mutants, and is not specific to defective axonal transport. However mutations in the *Khc* gene have been shown to exhibit a temperature-sensitive paralysis, a phenotype found in bang-sensitive paralytic (para) and *maleless* (*mle*) mutants, and interact with *para* and *mle* to cause lethality [62]. *Khc* mutants also suppressed the *Shaker* and *ether-a-go-go* leg shaking phenotype, which is similarly suppressed by *para* and *mle* alleles [62]. Hurd et al. conclude that Khc is likely required for anterograde transport of vesicles bearing sodium membrane channels and that reduced transport of these channels results in reduced sodium current. Likewise, the identification of a progressive bang-sensitive phenotype in the *stai* mutants we have analyzed, suggests that the transport of ion channels to the axon membrane is impaired.

Our observation of age-dependent phenotypes in *Drosophila* *stai* mutants parallels the observation that *STMN1* knockout mice initially develop normally and display no overt phenotypes [63], but when aged exhibit a minor axonopathy [64] and behavioral deficits that include a lack of both learned and innate fear responses [65]. Interestingly, altered levels of *STMN1* expression in human brain tissue has recently been linked to intractable temporal lobe epilepsy [66].

It has recently been reported that *stai* is required for stability of the neuromuscular junction in *Drosophila* , a phenotype that likely arises as a result of impaired axonal transport [67]. While Graf et al., present evidence of perturbed axonal transport, analysis of the integrity of axonal MTs did not reveal alterations in MT architecture. Our analysis supports the observation that *stai* function is necessary for axonal transport. In contrast to the studies of Graf et al., however, we also present evidence that the MT cytoskeleton of the axon is disrupted in *stai* mutant larvae and likely the cause of impaired axonal transport. We demonstrate this in three ways. First, we observe a reduction in the intensity of Futsch and acetylated α-tubulin staining in the segmental nerve axon that correlates with a reduction in *stai* dosage, and an increase in the severity of CSP accumulation in the axon. Second, we observe alterations in the organization of Futsch staining in individual segmental nerve axons lacking *stai* function. Finally, within the segmental nerve, individual axons exhibit an altered, distorted morphology in which they undulate along the length of the compound nerve bundle. We interpret this phenotype as a consequence of reduced axonal MTs that result in a loss of internal structural support for the axon. Interestingly, this phenotype is similar to the morphology of axons that are subjected to dynamic stretch injury, resulting in the breakage of MTs within the axon that correlate with undulations in axonal morphology [68]. One possible explanation for the observed differences in axonal MT morphology reported by each group is perdurance of maternally contributed stai product in differently aged third instar larvae examined in each study.

A wealth of evidence indicates that *stai* family members are negative regulators of MT dynamics. Decreasing the levels of *stai in vivo* increases polymerized MTs, as demonstrated by microinjection of neutralizing anti-stathmin antibody into newt lung cells [69], shRNA depletion of endogenous stathmin in human interphase cells [70,71], and knockout of stathmin function in MEFs [27], and amygdala slices from whole brains of stathmin knockout mice [65]. Conversely, microinjection of wildtype stathmin protein into newt lung cells results in massive loss of MT polymer [72]. Surprisingly, in contrast to vertebrate systems, loss of stai function results in dramatic reduction in the levels of both α-tubulin and acetylated α-tubulin. This reduction was visible on western blots as well as by immunohistochemical visualization that showed that polymerized MTs were reduced in our *stai* mutant *Drosophila* .

It is not clear why we see a reduction in the levels of polymerized tubulin in our *stai* mutant *Drosophila* . It has been previously demonstrated that the addition of recombinant *Drosophila* stai to purified bovine brain tubulin *in vitro* results in a decrease in MT polymerization in a dose dependent manner, while the expression of three different *Drosophila* *stai* isoforms, *staiA1*, *staiB1* and *staiB2* in HeLa cells *in vivo* depolymerizes the MT network in a subset of cells [22]. These observations indicate that *Drosophila* stai isoforms also act as MT depolymerizers in mammalian cells and mammalian cell extracts. It is possible that the MTs within the axonal compartment of a *Drosophila* nerve cell are subject to extrinsic signals, regulatory factors and MAPs that are distinct and not present in other subcellular regions of a neuron, or in different cell types. While this may be true, it is an unsatisfactory explanation because we also observe a reduction in polymerized MTs in muscle cells that comprise the body wall musculature.

Another possibility is that reduction in *stai* could alter the expression of a gene that encodes a MT polymer stabilizer. However, a more likely explanation for the observed reduction of the MT network in *stai* mutant *Drosophila* , is that the phenotype is the consequence of a complete depletion of all stai protein activity. Unlike vertebrate model systems, the entire *stai* family of proteins are encoded by a single gene in *Drosophila* that produces four stai protein isoforms [22]. Therefore, mutation in the *stai* gene removes or greatly reduces the activity of all stai proteins. In contrast, in studies performed in vertebrate model systems the function of only one member of the *stai* family is altered, and genetic redundancy by the remaining stathmin-like proteins may compensate and account for the observed phenotypic differences. Indeed, it has been reported in STMN1 knockout MEFs that the expression of STMN2 is decreased and STMN4 is increased [59], while in STMN1 knockout mice, an increase in the expression of STMN3 and STMN4 is observed [64,73]. The stai protein has two known activities; a MT depolymerizing activity as well as a tubulin binding activity. Depleting stai not only removes the MT depolymerizing activity of the protein, which is expected to result in an increase in the polymerized MT network, but it also abolishes its tubulin binding activity as well, which may be required to maintain the polymerized MT network. The net effect of removing both functions entirely may be a gross reduction in the MT network.

Observation of the MT network in muscle cells of third instar larvae may provide a clue as to how the MT network becomes reduced in *stai* mutant larvae. The distinct architecture allowed us to observe an abundance of fragmented or severed MTs in the peripheral cytoplasm of cells lacking *stai* activity. The MT cytoskeleton of a cell is subject to both stabilizing and destabilizing factors that act coordinately to maintain the MT architecture. For example, an antagonistic relationship in maintaining the MT network has been demonstrated between tubulin-specific chaperone E (TBCE), required for the formation of tubulin heterodimers, and the microtubule severing protein spastin [74]. It is possible that stai acts downstream of TBCE, and its removal results in a diminished MT network, as observed in TBCE mutant larvae, suggesting an antagonistic relationship with MT severing proteins spastin and/or katanin. It is interesting to note that mutation of TBCE in mice results in a progressive motor neuropathy [75,76] while mutations in spastin are the cause of the most common form of hereditary spastic paraplegia in humans, likely through disruption of axonal transport [12–14].

Stai is a cytosolic phosphoprotein whose function is regulated by many different signaling pathways [77]. The ability of stai to bind tubulin is dependent on its phosphorylation state. In *Xenopus* metaphase extracts, the phosphorylation of stathmin is in part regulated by the serine/threonine type-2A phosphatase PP2A [78]. PP2A is known to associate with MTs and is thought to regulate MTs through phosphorylation of the MAP Tau [79,80]. Interestingly, PP2A is a component of the striatin-interacting phosphatase and kinase (STRIPAK) complex, of which the Monopolar spindle-one-binder protein 3 (Mob3/Phocein) is a known component [81]. *DMob4*, the *Drosophila* Phocein homolog has recently been demonstrated to regulate microtubule organization and axonal transport [82].

Defects in axonal transport are known to cause or contribute to neurodegenerative disease and it is possible that *stai* may play a role in some cases. In mammals, loss of *stai* function results in mild, progressive phenotypes consistent with neurodegenerative disease [64]. In addition, a link has been established between the altered expression of stathmin and the abnormal architecture of MTs and organelle transport in motor axons of a mouse model of the motor neuron degeneration disorder spinal muscular atrophy [83]. Our *in vivo* analysis in *Drosophila* has identified a role for *stai* in regulating the architecture of MTs in the axon of peripheral nerves, necessary to support and sustain axonal transport.

## Supporting Information

Figure S1 Loss of stai Function Alters the Integrity of Muscle Microtubules. (**A**–**F**) Confocal micrographs obtained from body wall muscle 6 from third instar larvae immunostained with antibody against the α-tubulin (DM1A) (green) and counterstained with nucleic acid stain Syto24 (red). (**A**) Wild type animals have an extensive, well-defined MT network in muscle cells. (**B**) The MT architecture, however, is greatly reduced in density in *stai* ^*B200*^ mutant larvae. (**C**) The MT architecture is more severely reduced in *stai* ^*B200*^ */Df*(*2L*) *Exel6015* mutant larvae. Loss of *stai* function not only reduces the density of the MT architecture, but also results in fragmented MTs in the peripheral cytoplasm. (**D**) The MT architecture is reverted to wildtype in *stai* ^*excision*^, a precise excision allele derived from *stai* ^*B200*^. The reduced density of the MT cytoskeleton is ameliorated with the ubiquitous expression of *Drosophila staiB2* (**E**) or human *STMN1* (**F**). In panels A’-F' the scale bar = 20 µm, n = nerve. (**G**) The density of perinuclear MTs was quantified in third instar larvae body wall muscle. Results are normalized against wildtype and are presented as mean ± SD.
(TIF)

Movie S1 Wildtype *Drosophila* third instar larvae exhibit a normal crawling behavior. (MOV)

Movie S2
*Drosophila* third instar larvae homozygous for *stai* ^*rdtp*^ exhibit a robust 'tail flip' phenotype during crawling. (MOV)

Movie S3
*Drosophila* third instar larvae homozygous for *stai* ^*B200*^ exhibit a robust 'tail flip' phenotype during crawling. (MOV)

Movie S4
*Drosophila* third instar larvae heterozygous for *stai* ^*rdtp*^ and *stai* ^*B200*^ recapitulate the robust 'tail flip' phenotype during crawling, observed in larvae homozygous for each *stai* allele. (MOV)

Movie S5
*Drosophila* third instar larvae homozygous for *stai* ^*KO*^ [45] exhibit a robust 'tail flip' phenotype during crawling. (MOV)

Movie S6
*Drosophila* third instar larvae homozygous for *stai* ^*excision*^, a reversion allele generated from the precise excision of the mutagenic *piggyBac* element *PBac*{*5HPw^+^*} in *stai* ^*B200*^, exhibit a normal larval crawling behavior. (MOV)

Movie S7
*Drosophila* third instar larvae homozygous for *stai* ^*B200*^ and a single copy of the rescue transgene *tub-staiB2* exhibit a rescued crawling behavior. (MOV)

Movie S8
*Drosophila* third instar larvae homozygous for *stai* ^*B200*^ and a single copy of the rescue transgene *tub-STMN1* exhibit a rescued crawling behavior. (MOV)

Movie S9 Representative movie showing the bang-sensitive phenotype as observed in 42-day old *stai* ^*B200*^ adult *Drosophila* . (MOV)

Table S1 Penetrance of the Posterior Paralysis Phenotype Observed in Wandering Third Instar Larvae. The penetrance and severity of the posterior paralysis phenotype was scored and quantified by measuring the severity of the tai-flip, as determined by the angle that the tail was raised above the substrate on which the larvae crawled. If the tail was raised greater than 40^o^ above the horizontal crawling plane during the crawling cycle, larvae were scored as having a robust tail-flip. If the tail was raised less than 40^o^ above horizontal during the crawling cycle, larvae were scored as having a mild tail-flip. If larvae exhibited a normal crawling behavior, they were scored as having no tail-flip. The crawling behavior of at least one hundred larvae was analyzed, for a minimum of one minute each, for each genotype tested.
(XLSX)

Table S2 Percentage of Adult *Drosophila* Exhibiting a Bang-Sensitive Phenotype. Adult male *Drosophila* were assayed for paralytic behavior in response to mechanical stimulation at six different age periods post eclosion. In wild type controls, a paralysis in response to mechanical stimulation was observed with a very low penetrance of 1.5% at 42 days of age, and 3.3% at 56 days of age. This response is attributed to the general effects of aging on the adult nervous system. A pronounced bang-sensitive phenotype is observed in 20.9% of homozygous *stai* ^*B200*^ mutant animals tested at 21 days of age, increasing to 75.8% by 42 days of age and 91.7% at 56 days of age. The bang-sensitive phenotype is also observed at appreciable levels in *stai* ^*B200*^ */+* and *stai* ^*rdtp*^ */+* flies, but with a later onset than observed in *stai* ^*B200*^ homozygous adults. At 42 days of age, 25.2% of *stai* ^*B200*^ */+* and 18.0% of *stai* ^*rdtp*^ */+* adults exhibit a bang sensitive phenotype, increasing to 58.0% of *stai* ^*B200*^ */+* and 52.9% of *stai* ^*rdtp*^ */+* flies by 56 days. Ubiquitous expression of a rescuing transgene, *tub-staiB2*, delays the onset of the bang-sensitive response to mechanical stimulation to 24.6% of *stai* ^*B200*^ */+; tub-staiB2/+* animals aged 56 days. A minimal response to mechanical stimulation, not significantly different that wildtype, was observed in *stai* ^*rdtp*^ */+; tub-staiB2/+* animals aged 56 days. The *tub-staiB2* transgene also delayed and ameliorated the bang-sensitive phenotype in *stai* ^*B200*^ homozygous adults. The response to mechanical stimulation by genotype matched *stai* ^*excision*^ adults is not significantly different than the response of wild type control animals. A minimum of fifty adult *Drosophila* were tested at each age for each genotype analyzed.
(XLSX)

Table S3 Average Recovery Period of Adult *Drosophila* Exhibiting a Bang-Sensitive Phenotype. The average time required for recovery from a bang-sensitive paralysis induced by mechanical stimulation as described in the Materials and Methods was assayed in adult male *Drosophila* . Time is represented as average recovery time (secs) ± standard deviation (SD).
(XLSX)

Table S4 Average Pixel Intensity of Futsch Immunostained Segmental Nerve Axons of *Drosophila* Third Instar Larvae. The average pixel intensity of Futsch immunostained segmental nerve axons was determined as described in the Materials and Methods. Average pixel intensity is reported as mean ± S.D. in arbitrary units (a.u.) of fluorescence.
(XLSX)

Table S5 Average Pixel Intensity of Acetylated α-Tubulin Immunostained Segmental Nerve Axons of *Drosophila* Third Instar Larvae. The average pixel intensity of acetylated α-tubulin immunostained segmental nerve axons was determined as described in the Materials and Methods. Average pixel intensity is reported as mean ± S.D. in arbitrary units (a.u.) of fluorescence.
(XLSX)
