# Supplementary figures and images for: The Microtubule Regulatory Protein Stathmin Is Required to Maintain the Integrity of Axonal Microtubules in Drosophila
Source: PLoS One. 2013 Jun 26;8(6):e68324. doi: 10.1371/journal.pone.0068324 (PMC3694009; doi:10.1371/journal.pone.0068324)

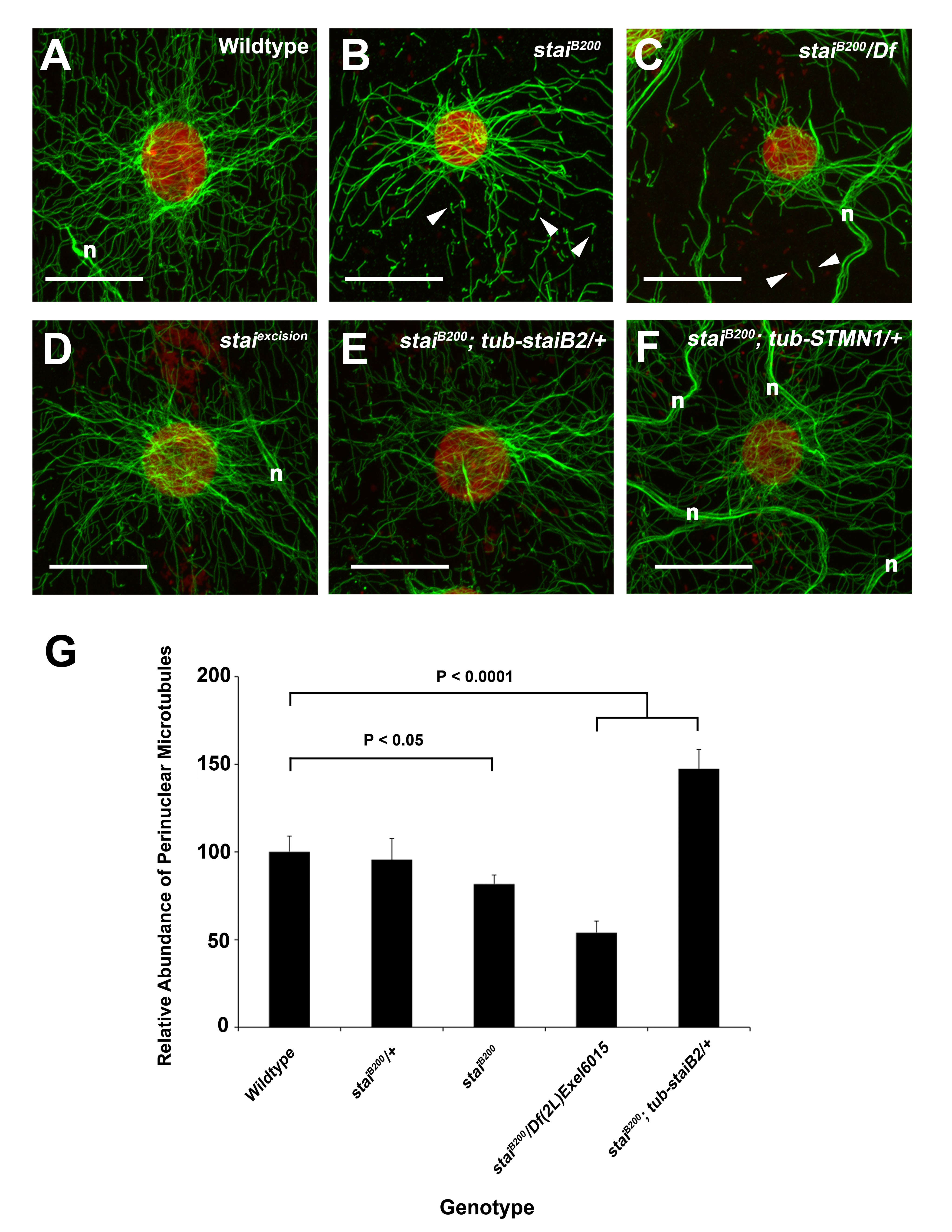

Supplement: Figure S1 — (A–F) Confocal micrographs obtained from body wall muscle 6 from third instar larvae immunostained with antibody against the α-tubulin (DM1A) (green) and counterstained with nucleic acid stain Syto24 (red). (A) Wild type animals have an extensive, well-defined MT network in muscle cells. (B) The MT architecture, however, is greatly reduced in density in stai B200 mutant larvae. (C) The MT architecture is more severely reduced in stai B200 /Df(2L) Exel6015 mutant larvae. Loss of stai function not only reduces the density of the MT architecture, but also results in fragmented MTs in the peripheral cytoplasm. (D) The MT architecture is reverted to wildtype in stai excision, a precise excision allele derived from stai B200. The reduced density of the MT cytoskeleton is ameliorated with the ubiquitous expression of Drosophila staiB2 (E) or human STMN1 (F). In panels A’-F' the scale bar = 20 µm, n = nerve. (G) The density of perinuclear MTs was quantified in third instar larvae body wall muscle. Results are normalized against wildtype and are presented as mean ± SD. (TIF) [file pone.0068324.s001.tif]
